# Structural changes correlated with magnetic spin state isomorphism in the S_2_ state of the Mn_4_CaO_5_ cluster in the oxygen-evolving complex of photosystem II†
†Electronic supplementary information (ESI) available. See DOI: 10.1039/c6sc00512h


**DOI:** 10.1039/c6sc00512h

**Published:** 2016-05-09

**Authors:** Ruchira Chatterjee, Guangye Han, Jan Kern, Sheraz Gul, Franklin D. Fuller, Anna Garachtchenko, Iris D. Young, Tsu-Chien Weng, Dennis Nordlund, Roberto Alonso-Mori, Uwe Bergmann, Dimosthenis Sokaras, Makoto Hatakeyama, Vittal K. Yachandra, Junko Yano

**Affiliations:** a Molecular Biophysics and Integrated Bioimaging Division , Lawrence Berkeley National Laboratory , MS 66-0200, 1 Cyclotron Rd. , Berkeley , CA 94720-8099 , USA . Email: JYano@lbl.gov ; Email: vkyachandra@lbl.gov ; Tel: +1 510 486 4366 ; Tel: +1 510 486 4963; b LCLS , SLAC National Accelerator Laboratory , Menlo Park , CA , USA; c Center for High Pressure Science &Technology Advanced Research , Shanghai , China; d SSRL , SLAC National Accelerator Laboratory , Menlo Park , CA , USA; e PULSE , SLAC National Accelerator Laboratory , Menlo Park , CA , USA; f RIKEN , Research Cluster for Innovation , Wako , Saitama , Japan

## Abstract

The Mn_4_CaO_5_ cluster in photosystem II catalyzes the four-electron redox reaction of water oxidation in natural photosynthesis. This catalytic reaction cycles through four intermediate states (S_*i*_, *i* = 0 to 4), involving changes in the redox state of the four Mn atoms in the cluster. Recent studies suggest the presence and importance of isomorphous structures within the same redox/intermediate S-state. It is highly likely that geometric and electronic structural flexibility play a role in the catalytic mechanism. Among the catalytic intermediates that have been identified experimentally thus far, there is clear evidence of such isomorphism in the S_2_ state, with a high-spin (5/2) (HS) and a low spin (1/2) (LS) form, identified and characterized by their distinct electron paramagnetic resonance (EPR spectroscopy) signals. We studied these two S_2_ isomers with Mn extended X-ray absorption fine structure (EXAFS) and absorption and emission spectroscopy (XANES/XES) to characterize the structural and electronic structural properties. The geometric and electronic structure of the HS and LS S_2_ states are different as determined using Mn EXAFS and XANES/XES, respectively. The Mn K-edge XANES and XES for the HS form are different from the LS and indicate a slightly lower positive charge on the Mn atoms compared to the LS form. Based on the EXAFS results which are clearly different, we propose possible structural differences between the two spin states. Such structural and magnetic redox-isomers if present at room temperature, will likely play a role in the mechanism for water-exchange/oxidation in photosynthesis.

## Abstract 2

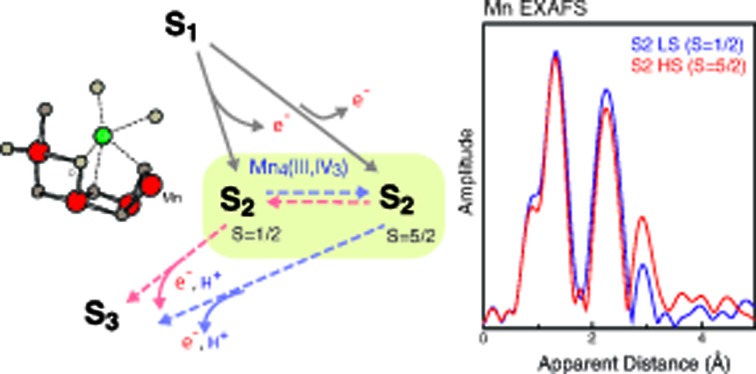
Mn XAS indicating different structures in the spin isomers of the S_2_ state of PSII

## Introduction

In oxygenic photosynthesis, light-driven water oxidation to molecular oxygen is carried out by the oxygen-evolving complex (OEC) in photosystem II (PSII). PSII is a multisubunit protein complex in the thylakoid membrane of plants, algae, and cyanobacteria.^1^,^2^ The OEC consists of four oxo-bridged Mn atoms and one Ca atom (Mn_4_CaO_5_) ligated to the D1 and CP43 subunits by carboxylate and histidine ligands.^3^,^4^ During water oxidation, the Mn_4_CaO_5_ complex cycles through five intermediate states, collectively called the S states, labeled S_0_–S_4_ in the Kok cycle.^5^ S_0_ is the most reduced state while S_1_, S_2_ and S_3_ represent sequentially higher oxidation states in the OEC. O_2_ is released during the S_3_ → [S_4_] → S_0_ transition, where S_4_ is a transient state. Thus, the Mn_4_CaO_5_ cluster accumulates four charges before the release of O_2_.

The oxidation state of each S-state has been formally assigned as MnIII3Mn^IV^ for S_0_, MnIII2MnIV2 for S_1_, Mn^III^MnIV3 for S_2_, and MnIV4 for S_3_.^6^–^10^ We note that there has been debate regarding the oxidation state assignment of the S_3_ state (*i.e.* whether it is formally MnIV4 or Mn^III^MnIV3 with charge delocalized on the ligands),^8^,^11^,^12^ and the current view from several experiments point more towards the formal oxidation state of MnIV4. However, formal oxidation states does not necessarily coincide with effective number of electrons in the metal valence shells because of important factors like metal–ligand covalency.^13^,^14^ A recent resonant inelastic X-ray scattering spectroscopy (RIXS) study indicate increasing delocalization of positive charge on to the ligands during the S-state transitions.^15^ Among the S-states, the S_2_ state is the most studied state due to the presence of rich EPR signals and nearly 100% conversion by illumination starting from the dark stable S_1_ state. The subsequent S_2_ to S_3_ state transition is accompanied by noticeable Mn–Mn distance changes,^16^ and several factors such as Ca-depletion,^17^ site-specific mutations,^18^ and chemical treatments (for example, with fluoride)^19^ are known to block this advance. The requirement for a structural change, and its susceptibility to many chemical and biochemical treatments, makes S_2_ to S_3_ transition one of the critical steps for water oxidation reaction during the S-state cycle.

In recent studies,^20^–^24^ the isomorphism observed in the S_2_ state has been suggested to be of importance in relation to the formation of the S_3_ state, where the chemical environment is prepared for the O–O bond formation to occur in the following steps. The presence of such chemical flexibility within the same OEC redox state (*i.e.* S-state) may play an important role in the catalytic process, for example, by providing a low energy barrier for the water exchange process. In the current study, we investigate isomorphism in the S_2_ state using Mn K-edge X-ray absorption, both XANES and EXAFS, and emission spectroscopy, and further discuss the mechanistic implication of such isomorphous states to the catalytic function of the OEC.

In the S_2_ state, two types of EPR signals have been assigned to the Mn cluster. The multiline signal (MLS) centered at *g* = 2 (S_2_-*g*2), exhibiting at least 18 partially resolved hyperfine lines at X-band (∼9 GHz), is a low spin (*S*_total_ = 1/2, *i.e.* Mn^III^/Mn^IV^ and Mn^IV^/Mn^IV^ are antiferromagnetically-coupled, respectively) ground state.^9^,^25^–^33^ Another broad featureless EPR signal at *g* ≥ 4.1 (S_2_-*g*4), attributed to a higher spin multiplicity (*S*_total_ = 5/2, *i.e.* ferromagnetically-coupled three Mn^IV^ with antiferromagnetically-coupled one Mn^III^) ground state, is also observed under different experimental conditions.^34^–^40^

**Scheme 1:**
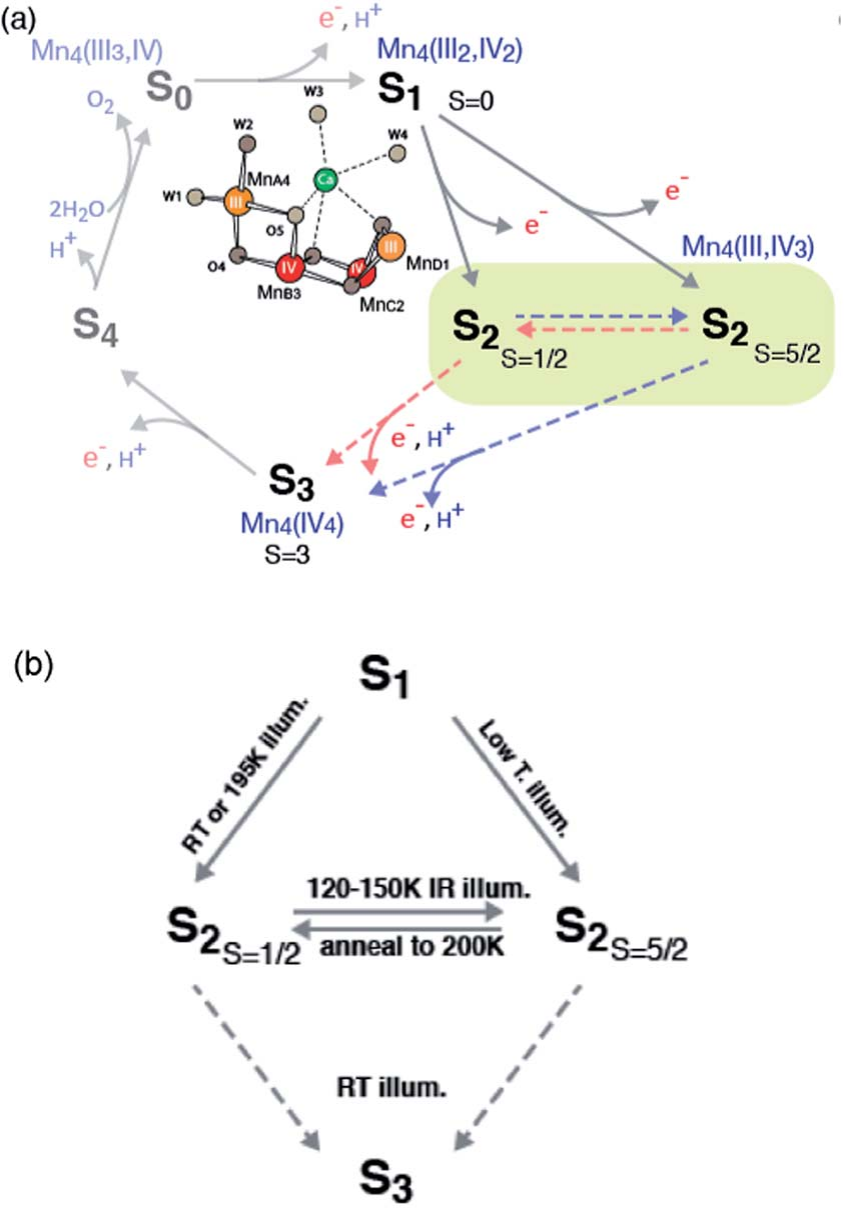
Formation of the S_2_-*g*2 and S_2_-*g*4 states in PSII from plants and the conversion between these two states.

The high spin (*S*_total_ = 5/2, called HS S_2_ or S_2_-*g*4 in the text) and low spin (*S*_total_ = 1/2, called LS S_2_ or S_2_-*g*2 in the text) forms in the S_2_ state are interrelated, on the basis of the observation of amplitude conversion of the S_2_-*g*4 EPR signal to the S_2_-*g*2 EPR signal.^34^,^41^–^43^ The distribution of high spin and low spin species, *g* values and hyperfine coupling values of these spin state changes are sensitive to several parameters, such as (a) species (higher-plant, thermophile or non-thermophile cyanobacterial PSII), (b) the presence of chemical additives like alcohol (methanol or ethanol), sucrose and glycerol (often used as a cryo-protectant) in the sample, (c) substitution of the native Ca^2+^ in the OEC (Ca^2+^-PSII) by Sr (Sr^2+^-PSII), and (d) halide substitution in PSII with Br^–^ or I^–^ replacing the Cl^–^ of the native state. A detailed discussion of these studies can be found in several reviews.^32^,^44^,^45^ Briefly, in samples illuminated at 195 K, both S_2_-*g*2 and S_2_-*g*4 signals are observed in the presence of sucrose, while with glycerol, ethylene glycol, or ethanol, the MLS is enhanced and the S_2_-*g*4 EPR signal is suppressed.^41^ Some treatments such as (c) and (d) stabilize the HS S_2_ form in the presence of the LS S_2_ form. Illumination by near-infrared (NIR) light at low temperature (∼150 K) has been shown to convert the S_2_-*g*2 form to the S_2_-*g*4 form without further advancement of S-state of the OEC.^39^,^41^ Subsequent annealing in the dark at 200 K converts the S_2_-*g*4 form back to the S_2_-*g*2 form,^34^ showing that these two forms are interconvertible. Both S_2_-*g*2 and S_2_-*g*4 forms show similar oscillation patterns around the S state cycle.^41^ PSII samples treated with NH_3_, F^–^, NO_3_^–^, or I^–^ or when Ca^2+^ is replaced by Sr^2+^ have been reported to show an enhanced S_2_-*g*4 signal with the line widths and *g* values being slightly different.^46^ The S-state transitions focused on the S_1_–S_2_–S_3_ steps are summarized in Scheme 1.

Recently, density functional theory (DFT) calculations by two groups suggest theoretical structural models corresponding to the two spin states^21^,^47^ and conclude that the two spin states are almost isoenergetic. *Ab initio* molecular dynamics simulations by Bovi *et al.* showed that these two states could interconvert over a low barrier (Δ*G*^#^ of 10.6 kcal mol^–1^).^48^ In proposed models by Pantazis *et al.*,^21^ the two spin states arise from a different location of Mn^III^; for LS S_2_, Mn^III^ is located in the corner of the cubane motif (Mn_D1_), while for HS S_2_, it is located at the tail Mn_A4_ (see Scheme 1). They along with a few other studies suggest that such isomorphism makes O_5_ unique, and that O_5_ may be a likely candidate for the slow-exchanging water in the S_2_ state.^3^,^4^,^22^,^49^–^54^

Previously, Liang *et al.* performed an XAS study on the HS S_2_ state.^55^ In their study, the authors concluded that HS S_2_ state is different from S_1_ and LS S_2_. They observe that the low spin S_2_ state showed a positive K edge shift compared to high spin state and an elongation of one of the Mn–Mn bond distances from 2.73 to 2.85 Å.^55^ In our current study, with improved data quality and the structural information available for the OEC S_1_ state from X-ray diffraction,^4^ we gain a detailed structural insight that will help us in understanding the mechanistic detail of the S_2_ to S_3_ transition.

In this study, we used X-ray absorption (XAS) and X-ray emission spectroscopy (XES) to study the nature of the two spin states in the S_2_ state. The possible structural changes are analysed based on the geometry obtained from the 1.95 Å resolution crystal structure of the S_1_ state.^4^ We discuss the structural and electronic structural differences of the two spin states, and its relation to the functional role in S_2_ to S_3_ transition and subsequently during the water oxidation reaction.

## Materials and method

### Preparation of PSII membranes

PSII-enriched membrane fragments were prepared under dim green light from spinach leaves according to Berthold *et al.*^56^ PSII membranes were resuspended to a chlorophyll (Chl) concentration of 8 mg Chl per mL in a buffer containing 30% (v/v) glycerol, 50 mM MES–NaOH (pH 6.0), 5 mM MgCl_2_, 5 mM CaCl_2_, 15 mM NaCl and stored at –80 °C until used. All samples were measured in this buffer. Oxygen-evolution activity of 400–500 μmol of O_2_ per mg of Chl per h was observed. The oxygen-evolution activity was measured in a buffer with 50 mM MES–NaOH (pH 6.0), 10 mM MgCl_2_, 5 mM CaCl_2_ and 15 mM NaCl at 25 °C under saturating light and in the presence of 0.5 mM phenyl-*p*-benzoquinone (pPBQ) as electron acceptor.

The samples for X-ray studies were prepared by mounting PSII membranes pellets (chlorophyll concentrations in these samples ranged from 20 to 25 mg mL^–1^) directly onto the Lucite sample holders, with a hollowed compartment (dimensions of 2.1 × 0.3 × 0.15 cm) backed by a piece of mylar tape. All illuminations, EPR, and X-ray measurements were performed directly on samples mounted in these holders.

### Generation of the S-states by illumination

All the sample preparations as described above were performed in the dark or with dim green light at 4 °C to poise the PSII centers in the S_1_ state and then the samples were frozen in liquid nitrogen. The HS S_2_ and LS S_2_ states were generated by light illumination at 140 ± 1 K or 195 K. Prior to illumination, dark-adapted samples were equilibrated for 3 min at 140 ± 1 K or 195 K. For 195 K illumination, the temperature was maintained in a dry ice/ethanol bath in an unsilvered dewar, and samples were continuously illuminated for 10 min using a 400 W tungsten lamp, with a 7 cm path of 5% CuSO_4_ as a heat and IR light filter. For 140 K illumination, the temperature was maintained with a continuous stream of liquid nitrogen-cooled nitrogen gas. Samples were continuously illuminated for 10 min using a 400 W tungsten lamp, with a 7 cm path of water as a heat filter. The temperature was monitored throughout the illumination period with a copper–constantan thermocouple. After illumination, samples were frozen in liquid nitrogen within 1–2 seconds. The 2 flash data used in this study was collected previously. The S_3_ spectra were deconvoluted using the protocol established previously.^57^

### EPR spectroscopy

Low-temperature X-band EPR spectra were recorded using a Varian E109 EPR spectrometer equipped with a Model 102 Microwave bridge. Sample temperature was maintained at 8 K using an Air Products LTR liquid helium cryostat. The following spectrometer conditions were used: microwave frequency, 9.22 GHz; field modulation amplitude, 32 G at 100 kHz; microwave power, 20 mW. The EPR signals were quantitated by adding the peak-to-trough amplitudes of S_2_-*g*4 or four of the downfield hyperfine lines of the S_2_-*g*2 MLS, respectively.

### XAS measurements

X-ray absorption spectra were collected at the Stanford Synchrotron Radiation Lightsource (SSRL) on beamline 7-3 at an electron energy of 3.0 GeV and an average current of 500 mA. The intensity of the incident X-rays was monitored by a N_2_-filled ion chamber (I_0_) in front of the sample. The slit in front of the I_0_ detector was closed to a vertical size of 2.5 mm and a horizontal size of 14 mm. The radiation was monochromatized by a Si (220) double-crystal monochromator. The total photon flux on the sample was limited to ∼3 × 10^6^ photons per μm^2^, which was determined to be non-damaging on the basis of detailed radiation-damage studies.^16^,^58^–^60^ We compared consecutive XAS scans from each sample and detected no shift in the K-edge energy over first five scans at each spot (Fig. S1†). The samples were protected from the beam during the monochromator movements between different energy positions by a shutter that was synchronized with the scan program. The samples were kept at 8 K in a He atmosphere at ambient pressure by using an Oxford CF-1208 continuous-flow liquid He cryostat. Data were recorded as fluorescence excitation spectra by using a germanium 30-element energy-resolving detector (Canberra Electronics). For Mn XAS, energy was calibrated by the pre-edge peak of KMnO_4_ (6543.3 eV), which was placed between two N_2_-filled ionization chambers (I_1_ and I_2_) after the sample.

Data reduction of the EXAFS spectra was performed using SIXPAK.^61^ Pre-edge and post-edge backgrounds were subtracted from the XAS spectra, and the results were normalized with respect to edge height. Background removal in *k*-space was achieved through a five-domain cubic spline. Curve fitting was performed with Artemis and IFEFFIT software using *ab initio*-calculated phases and amplitudes from the program FEFF 8.2.^62^,^63^ EXAFS curve-fitting procedure is described in detail in the ESI.† Mn XANES pre-edge spectra were fit using EDG_FIT in EXAFSPAK.^64^ The XANES inflection point energy (IPE) was extracted from zero crossing of the second derivative in the energy region between 6550 eV and 6554 eV.

### XES measurements

X-ray emission spectra were collected at SSRL on beamline 6-2. The beamline monochromator, using two cryogenically cooled Si crystals in (111) reflection, was used to set the incident photon energy to 10.4 keV. The X-ray beam was focused to 0.45 (V) × 0.45 (H) mm (fwhm) by means of vertical and horizontal focusing mirrors. The X-ray flux at 10.4 keV was ∼1 × 10^13^ photons per s per mm^2^. During the measurement, samples were kept at 10 K in a continuous flow liquid helium cryostat (Oxford Instruments CF1208) under helium exchange gas atmosphere. Emission spectra were recorded by means of a high-resolution crystal-array spectrometer, using the 440 reflection of 7 spherically bent Si(110) crystals (100 mm diameter, 1 m radius of curvature), aligned on intersecting Rowland circles.^65^ An energy-resolving Si drift detector (Vortex) was positioned at the focus of the 7 diffracting elements. A helium-filled polyethylene bag was placed between the cryostat and the spectrometer to minimize signal attenuation due to air absorption. Each energy point in the spectra was collected at a fresh sample spot. The maximum exposure time at each spot was 2.5 seconds and the signal was read out in bins of 50 ms duration. At first, a time-scan at a single emission energy was carried out for each S-state to check the onset time of radiation-induced changes of the signal intensity. No changes were observed at least for the first 1.5 s, and therefore the first 20 bins (equivalent to 1 s) were averaged for the final spectra. The signal intensity from each sample spot was normalized by the emission signal intensity recorded at 6491.5 eV within 7 s from the same sample spot, after going through all the fresh spots. Fig. S2† shows the XES spectra after first 20 bins (equivalent to 1 s) and from bin 11–30 (0.5–1.5 s) of the 140 K NIR illuminated sample. We see no damage till 1.5 s of data collection.

### Computational details

The optimizations were carried out using Gaussian 09 (ref. 66) and ONIOM calculation.^67^ The initial structure was based on a previous study on the OEC in the S_2_ state.^68^*S* = 13/2 spin state was used so that the oxidation states of Mn ions in the S_2_ state is Mn^III^MnIV3. The Mn oxidation states were determined by the Mulliken's spin population analysis. The high layer of ONIOM calculation was assigned to the Mn_4_CaO_5_ cluster and the ligands (Asp170, Glu189, His332, Glu333, His337, Asp342, Ala344, Glu354, Arg357, W1–W4 and other water ligands). Notations for each residue are similar to those in the PDB-data (; 3ARC).^3^ The low layer of the ONIOM was assigned to the residues within 40 Å radius of Ca in the Mn_4_CaO_5_ cluster. The high layer was calculated with wB97XD DFT functional,^69^ LanL2DZ basis sets for metals (Mn, Ca) and 6-31G(d) for other atoms (H, C, N, O). The low layer was calculated with Amber force field.^70^

## Results

### EPR characterization

**Fig. 1:**
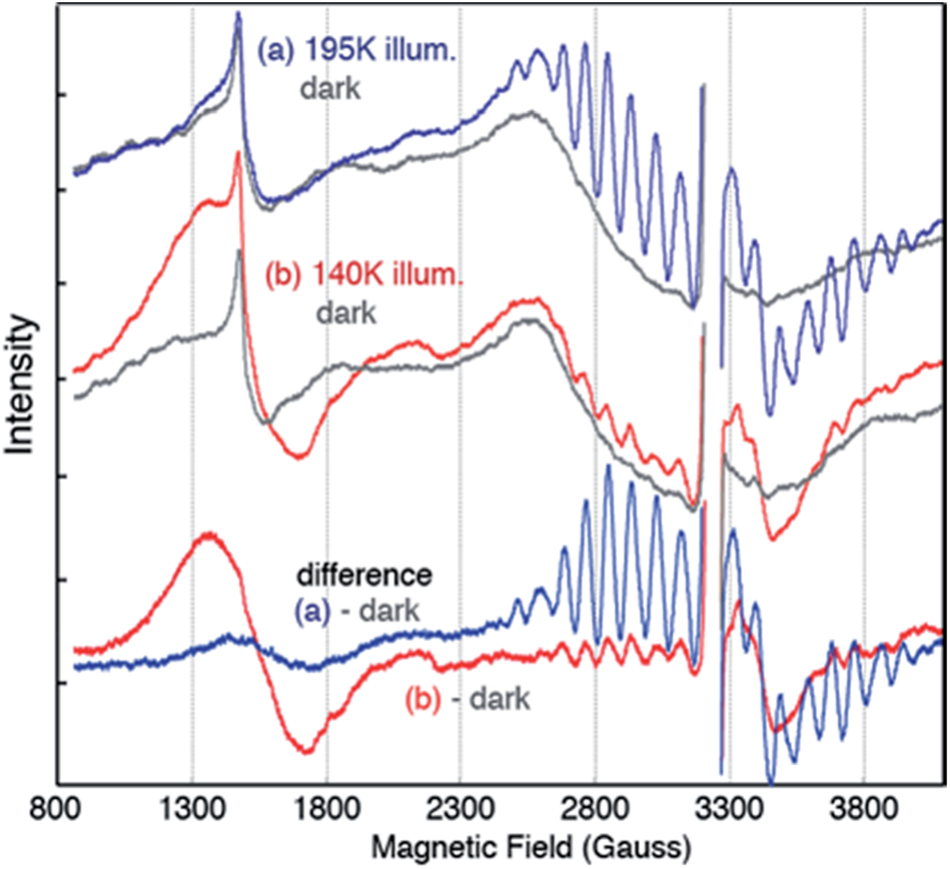
EPR spectra of PSII samples in glycerol illuminated for 10 minutes at (a) 195 K (blue) (b) 140 K with NIR (red) along with corresponding dark (grey) EPR spectra. The difference spectra are between the spectra after illumination and the spectra of the same dark-adapted sample. The large intensity from Y_D_˙ in each spectrum has been removed for clarity (∼3200 G). Spectrometer condition: microwave frequency, 9.22 GHz; field modulation amplitude, 32 G at 100 KHz; microwave power, 20 mW. The spectra are collected at 8 K.

**Fig. 2:**
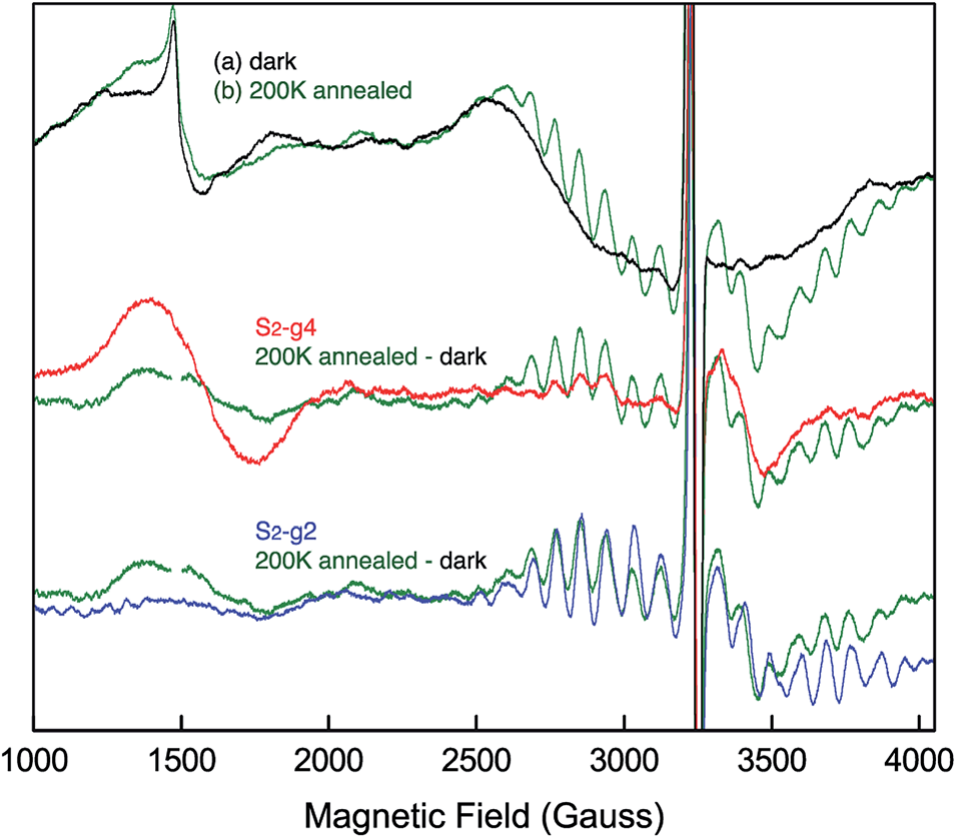
EPR spectral changes of the sample annealed at 200 K (green) after 140 K NIR illumination. The difference spectrum between annealed sample (green) and dark is compared to the 140 K NIR illuminated minus dark or S_2_-*g*4 (red) and 195 K illuminated minus dark or S_2_-*g*2 (blue) samples. The spectra are collected at 8 K. Spectrometer condition: microwave frequency, 9.22 GHz; field modulation amplitude, 32 G at 100 KHz; microwave power, 20 mW.

EPR spectra from the spinach PSII S_2_ states in 30% glycerol buffer are shown in Fig. 1. Illumination of PSII membranes at 195 K results in the formation of the S_2_ MLS. Under these illumination conditions, the dominant feature is the S_2_ MLS that corresponds to the total spin (*S*_total_) of 1/2 that arises from exchange interaction of one high-spin Mn^III^ and three high-spin Mn^IV^, as has been intensively studied in the past.^9^,^25^–^33^ While a weak, broad peak is also present in the region around *g* = 4 (Fig. 1 ((a) minus dark)), the small intensity of the signal shows that this species is nearly absent under our experimental conditions. When PSII membrane samples are illuminated at low temperature (140 K) in the absence of an IR filter, the photogeneration of the broad S_2_-*g*4 signal is observed, with a small S_2_-*g*2 signal (Fig. 1 ((b) minus dark)). The amount of the S_2_-*g*2 in the sample illuminated at 140 K is approximately 20% of the intensity of S_2_-*g*2 signal from 195 K illuminated sample. The transition from the HS S_2_ to LS S_2_ occurs by increasing the temperature, which is supported by the reduction of the S_2_-*g*4 EPR signal and the increase of the S_2_-*g*2 signal when the 140 K NIR illuminated sample (S_2_-*g*4 dominant) is annealed to 200 K. To shows that there is interconversion between the S_2_-*g*4 and S_2_-*g*2 species by temperature, EPR data of the annealed sample were collected at 8 K. The S_2_-*g*2 signal of the annealed sample increased up to 70% level of the 195 K illuminated sample while the S_2_-*g*4 signal decreases down to ∼30% (Fig. 2).

### O_2_ activity of the *g*2 rich and *g*4 rich spinach PSII

It is known that PSII samples from spinach in the S_2_ state in glycerol buffer have a dominant S_2_-*g*2 signal with only a trace of the S_2_-*g*4 signal for 195 K illumination, while the PSII in sucrose buffer have both S_2_-*g*2 signal and S_2_-*g*4 signal in almost 50 : 50 ratio.^41^ We observe a similar trend in the EPR spectra of these samples are shown in Fig. 1 (glycerol) and Fig. S3 (sucrose) in the ESI.† To check the activity of the S_2_-*g*4-rich and S_2_-*g*2-rich PSII samples, the O_2_ evolution activity of both samples are compared by dividing the same batch of PSII thylakoid samples into two parts and transferring one part into glycerol buffer, and the other into sucrose buffer [50 mM MES–NaOH (pH 6.0), 5 mM MgCl_2_, 5 mM CaCl_2_ and 15 mM NaCl, 0.4 M sucrose]. The O_2_ activity was very similar between the two samples, giving rates of 420 ± 10 μmol of O_2_ per mg of Chl per h in glycerol buffer and 408 ± 10 μmol of O_2_ per mg of Chl per h in sucrose buffer. These measurements were performed with three different sample preparations. The results shows that the number of the active centers is more or less the same in the two samples, while the fraction of centers that can be cryo-trapped in the S_2_-*g*4 or the S_2_-*g*2 spin states is significantly different, depending on the buffer conditions.

### Mn K-edge spectra

**Fig. 3:**
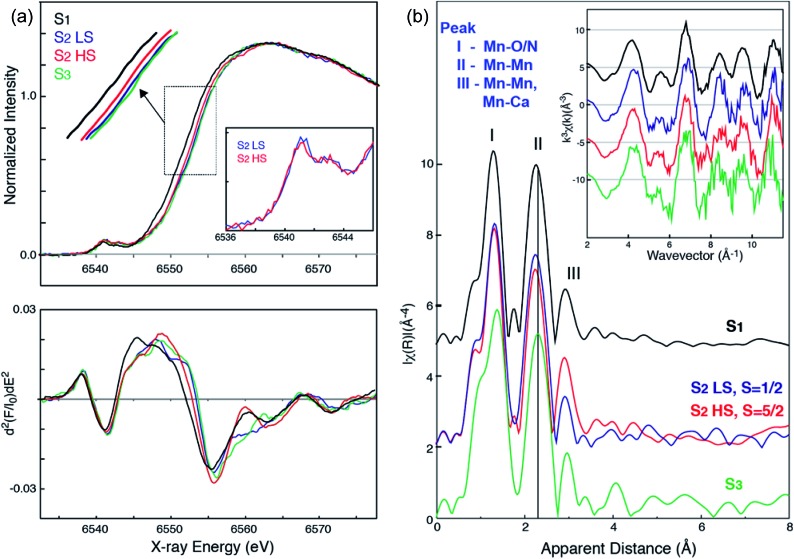
(a) Mn XANES spectra (top) and their second derivative spectra (bottom) of HS (red) and LS (blue) S_2_ states, in comparison with S_1_ (black) and S_3_ (green) states. Mn preedge of HS (red) and LS (blue) S_2_ states (inset). (b) Mn EXAFS spectra of HS (red) and LS (blue) S_2_ states, in comparison with S_1_ (black) and S_3_ (green) states. *k*^3^-Weighted EXAFS spectra (inset) and their Fourier-transformed spectra of HS (red) and LS (blue) S_2_ states, in comparison with S_1_ (black) and S_3_ (green) states are shown. Prominent changes in peak II of the FT spectra between the different states are indicated by a line (black). All spectra are shown in the same scale but with a vertical offset. Pure (deconvoluted) S-states are shown in the figure.

Fig. 3a shows the Mn XANES spectra of the two S_2_ spin states (HS S_2_ and LS S_2_), together with S_1_ and S_3_ states. In the presence of glycerol as a cryo-protectant, the majority of the PSII centers are in the LS S_2_ state when illuminated at 195 K, as observed in the EPR spectra (see Fig. 1 ((a) minus dark)). On the other hand, illumination at 140 K generates a large fraction of the PSII centers in the HS S_2_ state. Using the estimated ratio of S_2_-*g*2 MLS intensity between the samples under the two illumination conditions (195 K illuminated *vs.* the 140 K illuminated samples), it is inferred that a minor fraction (∼20%) of LS S_2_ state is present in the samples illuminated at 140 K. The corresponding amount of LS S_2_ state spectrum was subtracted from the XAS spectrum of 140 K NIR illuminated sample to obtain the pure HS S_2_ XAS spectrum. This is based on the assumption that 195 K illuminated and 140 K NIR illuminated samples consist of a linear combination of the HS and LS S_2_ states. The pure LS S_2_ XAS spectra were also obtained by the same method. The untreated XAS spectra from the 195 K illuminated and the 140 K NIR illuminated samples are shown in Fig. S4 in the ESI.†

Interestingly, the XANES rising edge position of the HS S_2_ state is slightly but noticeably lower in energy than that of the LS S_2_ state as shown in Fig. 3a. While the edge positions of LS S_2_ and S_3_ states are very close, their spectral shapes are not exactly the same. This difference is more clearly seen in the 2^nd^ derivative spectra (Fig. 3a bottom). The inflection point energy obtained from the 2^nd^ derivative XANES spectra are, 6552.11 eV (S_1_), 6552.89 (S_2_ HS), 6553.44 (S_2_ LS), and 6553.71 (S_3_). It is often difficult to compare these numbers with literature values due to different procedures for generation of the 2^nd^ derivative spectra. Therefore, we compared XANES spectra of all S-states treated in the same way, to eliminate any ambiguity that may arise from such data treatment. We further note that the inflection point energy could be a possible indicator of Mn charge density, although multiple-scattering effects in the XANES region could mask such changes when the structural changes are accompanied by oxidation state changes. For this reason, we cautiously state that the edge shift observed in the HS and LS S_2_ state suggests a change in charge density of Mn in these two states. The HS S_2_ state might be slightly lower in the effective positive charge density on Mn compared to the LS S_2_ state. We confirmed that there is no indication of Mn^II^ being released during the 140 K NIR illumination by monitoring the presence of the Mn^II^ EPR signal since such an effect will also lower the Mn XANES edge position. Another potential cause for lowering the Mn edge position is the presence of a fraction of the S_1_ state in the HS S_2_ sample due to the low temp. illumination. We excluded this possibility based on the results of the annealing experiments (Fig. 2). After annealing the S_2_-*g*4 sample to 200 K, the multiline (*g* = 2) spectra increased to 70% compared to the S_2_-*g*2 state spectra. On the other hand, the *g* = 4 part of the annealed spectra was reduced to 30% of the S_2_-*g*4 spectra.

In addition, we investigated the Mn XANES pre-edge peaks of the two states, as it serves as another indicator of the effective charge density. The pre-edge spectra are slightly, but noticeably different in the LS S_2_ and HS S_2_ states (Fig. 3a inset). The pre-edge spectra were fit with a pseudo-Voigt line with a 1 : 1 ratio of Lorentzian and Gaussian functions and the peak area was compared between these two states. The number of the pre-edge components and their positions were estimated by the 2^nd^ derivative spectra. The area of the pre-edge peak was 0.22 for LS S_2_ and 0.24 for HS S_2_ (Fig. S5 and Table S1 in the ESI†).^71^ While the slightly larger pre-edge area observed in HS S_2_ may indicate a more distorted ligand environment in this state as compared to LS S_2_, the difference is rather small for drawing any concrete conclusions.

Fig. 3b shows the EXAFS spectra of the two S_2_ spin states, together with the S_1_ and S_3_ state spectra. A comparison of the HS and LS S_2_ state spectra shows noticeable differences in the 2^nd^ Fourier transform (FT) peak width and intensity as well as the 3^rd^ FT peak intensity, which is significantly higher in the HS S_2_ state spectrum. The 2^nd^ FT peak corresponds to the di-μ-oxo bridged Mn–Mn interactions around 2.7 Å and the 3^rd^ FT peak arises from the contribution of mono-μ-oxo bridged Mn–Mn and Mn–Ca interactions around 3.3 Å. Such differences are also visible in the EXAFS oscillation in the *k*-space spectra (Fig. 3b inset). Furthermore, both HS and LS S_2_ spectra differ from the S_3_ state spectrum, suggesting that the structural geometries in these three states are not the same. Detailed EXAFS analysis is discussed in the next section.

### Mn EXAFS curve fitting

**Fig. 4:**
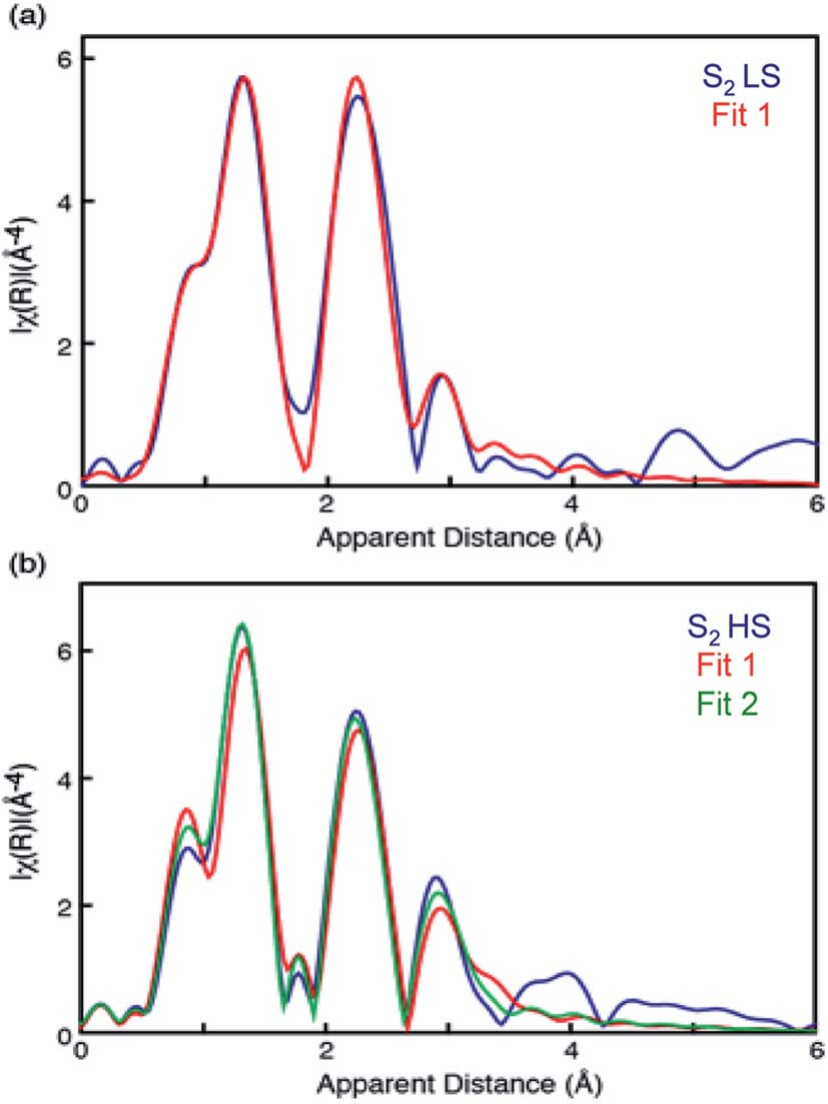
EXAFS curve fitting results of HS (bottom) and LS (top) S_2_ states. The parameters are listed in Table 1.

Mn EXAFS curve fitting of the HS and LS S_2_ states were carried out to extract structural parameters of the Mn cluster in these states. Descriptions of the parameters used are provided in the ESI.† Fig. 4 shows fit results, and the fit parameters are summarized in Table 1. Structures for LS and HS S_2_ state have been proposed previously based on EPR and quantum chemical calculations,^21^ and we therefore used those as starting structural models for fitting the EXAFS data.

**Table 1:** Mn EXAFS curve fitting parameters for the HS and LS S_2_ species^*a*^

Sample	Fit #	Shell	*N*	*R*/Å	*σ* ^2^/Å	*R*/%
LS S_2_	1	Mn–O	4	1.83 (0.01)	0.005 (0.001)	4.2
		Mn–O(N)	2	2.00 (0.03)	0.004 (0.001)	
		Mn–Mn	1.5	2.72 (0.02)	0.002 (0.002)	
		Mn–Mn	0.5	3.24 (0.11)	0.005 (0.001)	
		Mn–Ca	0.75	3.36 (0.01)	0.007 (0.001)	
		Mn–Ca	0.25	3.89 (0.01)	0.005 (0.001)	
					*E*_0_ (eV) = –9.3	
HS S_2_	1	Mn–O	4	1.85 (0.03)	0.005 (0.001)	2.6
		Mn–O(N)	2	2.05 (0.07)	0.006 (0.006)	
		Mn–Mn	1.5	2.74 (0.03)	0.004 (0.003)	
		Mn–Mn	0.5	3.30 (0.07)	0.001 (0.001)	
		Mn–Ca	0.75	3.36 (0.01)	0.007 (0.001)	
		Mn–Ca	0.25	4.09 (0.01)	0.015 (0.001)	
		Mn–C	3	4.39 (0.17)	0.002 (0.001)	
					*E*_0_ (eV) = –8.1	
	2	Mn–O	4	1.84 (0.03)	0.005 (0.003)	1.2
		Mn–O(N)	2	2.02 (0.06)	0.007 (0.008)	
		Mn–Mn	1	2.72 (0.02)	0.001 (0.001)	
		Mn–Mn	1	3.30 (0.05)	0.005 (0.001)	
		Mn–Ca	0.75	3.26 (0.14)	0.007 (0.001)	
		Mn–Ca	0.25	4.09 (0.01)	0.006 (0.001)	
		Mn–C	3	4.35 (0.14)	0.004 (0.001)	
					*E*_0_ (eV) = –11.2	

^*a*^S_0_^2^ was set to 0.85. *σ*^2^ is the Debye–Waller factor, *R* (%) shows the goodness of fit. The fit range of all the spectra are *k* = 2.4–11.3 Å^–1^ (*R* = 1–4.2 Å). Note that for Mn–Mn interactions, *N* = 1.5 implies that there are three similar interactions within the cluster, as the number of interactions is divided by the number of Mn in the cluster (*i.e.* 3/4 = 1.5) to be normalized it to per Mn. Similarly, Mn–Ca 0.5 means there are two similar Mn–Ca interactions within the cluster (*i.e.* 2/4 = 0.5).

**Scheme 2:**
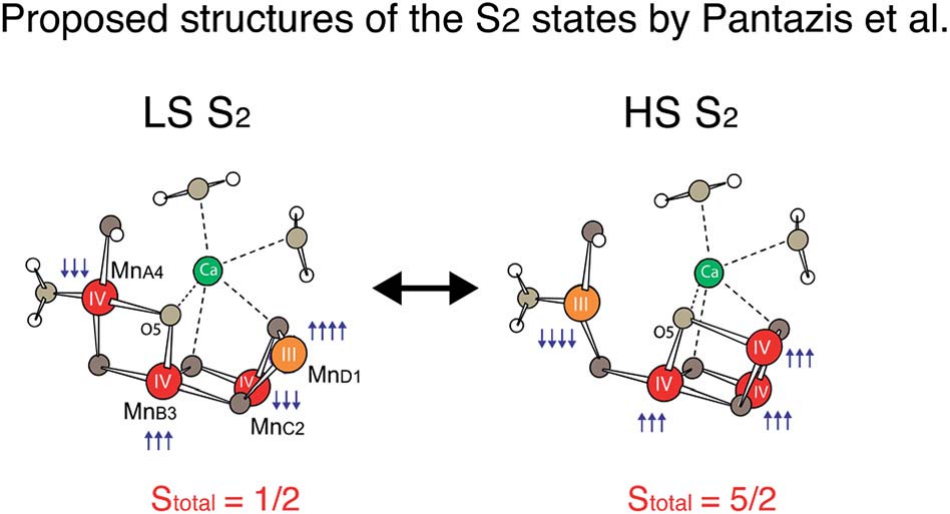
Proposed HS and LS S_2_ state structures by Pantazis *et al.*^21^ as well as Isobe *et al.*^47^

The LS S_2_ state fits well with the proposed open cubane-like structure.^16^,^21^ In this structure, there are three short Mn–Mn interactions around 2.7–2.8 Å and one long Mn–Mn interaction around 3.3 Å (LS S_2_ – fit #1 in Fig. 4 and Table 1). For the HS S_2_ state, the left-open structural model was suggested by Pantazis *et al.* and Isobe *et al.* from QM/MM calculations (Scheme 2).^21^,^47^ In this model, the numbers of short and long Mn–Mn interactions and Mn–Ca interactions in the HS S_2_ state remain the same as in the LS S_2_ model. Therefore, the same parameters obtained from the LS S_2_ fit (LS S_2_ – fit #1) were used as starting parameters (HS S_2_ – fit #1). In the experimental spectrum of HS S_2_, the 3rd FT peak intensity increases noticeably, while the 2nd FT peak becomes narrower than that of the S_2_ LS spectrum. In HS S_2_ (fit #1), the atomic distances remained similar to the initial parameters. The weaker FT peak II intensity and the stronger FT peak III intensity were compensated by Debye Waller factors. While FT peak II could be fit with three Mn–Mn interactions with an average distance of 2.73 Å, the presence of a longer ∼2.8 Å Mn–Mn interaction was not preferable. This observation suggests that a complete cubane with Mn_D1_, Mn_C2_, Mn_B3_, and Ca cannot be formed in this state; as such a structure typically will have high distance heterogeneity in the range of 2.7 to 2.8 Å. We also tested a hypothetical model where one of the three Mn–Mn short distances elongates, as it would in the presence of a mono-μ-oxo-like bridge, *i.e.* giving a short (2.7–2.8 Å) and long (∼3.3 Å) Mn–Mn distances ratio of 1 : 1 (HS S_2_ – fit #2). The fit quality was improved by 50% for this model. In a later section, we further discuss (a) whether such a structure is possible, (b) the interconversion between HS and LS form in the S_2_ state, and (c) the relation of the two S_2_ isomers to the formation of the S_3_ state.

### Mn Kβ_1,3_/Kβ′ XES

**Fig. 5:**
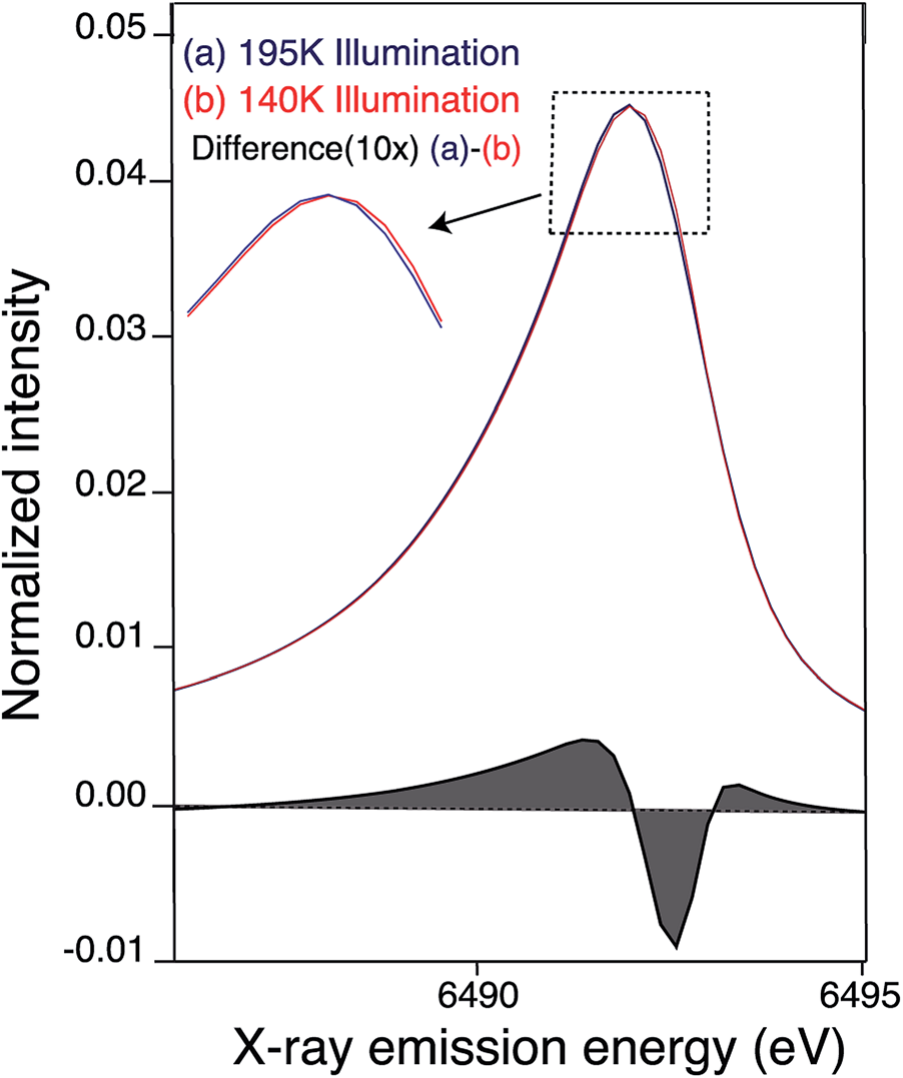
Kβ_1,3_ emission (smoothed) spectra of S_2_ sample obtained by (a) 195 K (blue) (b) 140 K NIR illumination (red). Difference between the Mn Kβ_1,3_ XES of PS II (at 10× enlargement) 195 illuminated – 140 K NIR illuminated S_2_ state is shown in black. The 195 K emission spectra from Fig. S6† was smoothed by fitting a sum of two asymmetric orthogonal lineshape functions. The 140 K spectrum is fit by a superposition of the 195 K fit and additional orthogonal lineshape functions. In order to obtain an unstructured residual a single additional orthogonal lineshape function is adequate, but significant structure is present in the residual if no additional lines are added. The minor fraction of the other state (HS S_2_ in case of LS S_2_ and *vice versa*) present is not subtracted in the spectra presented here.

XES Kβ_1,3_/Kβ′ transitions provide complementary information to XANES, by probing the Mn 3p to 1s emission process that is sensitive to the number of unpaired 3d electrons through 3d/3p spin exchange interactions. We measured the Kβ_1,3_ XES spectra of the LS and HS S_2_ states. We observe a slight shift in the Kβ_1,3_ emission spectra between the LS and HS S_2_ states (Fig. 5). Fig. S6† shows raw and smoothed data for the Kβ_1,3_/Kβ′ XES transitions for LS and HS S_2_ states along with the residual plot. The spectra were smoothed using a sum of nonlinear lineshapes. We observe that the spectrum of the LS S_2_ state is at a slightly lower energy than the HS S_2_ state, as becomes evident in the difference spectra of the LS and HS S_2_ states (Fig. 5). With an increase in the oxidation state of Mn, fewer unpaired 3d valence electrons can interact with the 3p hole, leading to a decrease in the magnitude of 3p–3d exchange interaction, which results in the Kβ_1,3_ emission spectra shifting to a lower energy. Hence, the LS S_2_ state might have slightly higher effective positive charge density on Mn compared to the HS S_2_. This is in agreement with the changes observed in the XANES spectra reported in the earlier section.

## Discussion

The nature of the two isomers in the S_2_ state of higher plant PSII was investigated using X-ray spectroscopy with the support of EPR spectroscopy. We have observed the XAS (XANES and EXAFS) and XES spectral changes between the HS and LS S_2_ species, and here we discuss possible structural models and the transition phenomena, with a comparison to the proposed models in the literature.

First, the fact that the population of the LS and HS S_2_ species seems to shift depending on the buffer conditions implies that some variation of the XANES edge positions for the S_2_ state shown in the literature may contain this effect since this kind of isomorphism was known but not differentiated until recently. Nevertheless, the LS S_2_ state should be the dominant spin state in the literature studies, when glycerol buffer is used.^7^,^11^,^16^,^72^

### Structural models of the high-spin and low-spin S_2_ states

**Fig. 6:**
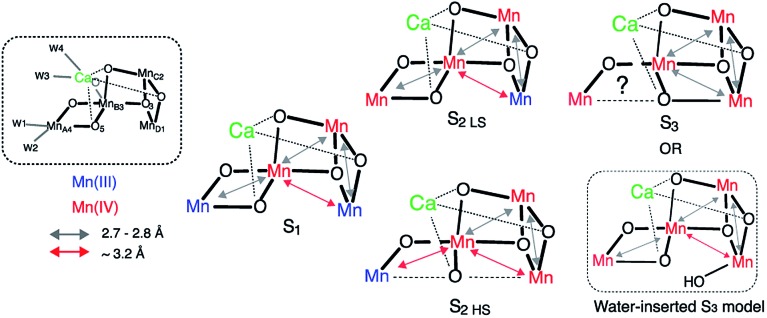
Proposed distance changes during the S_1_–S_2_–S_3_ transitions, based on the current EXAFS data and proposed models by Cox *et al.* and Siegbahn *et al.*^12^,^20^,^52^,^60^

The differences observed in the XAS spectra provide evidence for the different electronic structure and the metal–metal atomic distances in the HS and LS S_2_ states. The HS and LS structural models that involve interconversion of the Mn^III^ position in these two species have been proposed by Pantazis *et al.*^21^ and Isobe *et al.*^47^ based on EPR results and from quantum chemical calculations. In the LS S_2_, Mn^III^ is located at the Mn_D1_ position that is ligated to His332 of D1 chain, while in the HS S_2_ state Mn^III^ is at the Mn_A4_ position of the cluster, which has two water ligands (Fig. S7†). The EXAFS curve fitting results match with the result that the LS S_2_ state is an open cubane-like structure in which Mn^III^ is at the Mn_D1_ site, as previously suggested. While in general EXAFS is not a technique that can be used to conclusively point to a single, unique, structural model,^73^ the observed peak intensity change in the FT EXAFS spectra is clear evidence of the structural differences between the two spin states. The EXAFS curve fitting results suggests that the ratio of short and long Mn–Mn interactions may be different in the HS and LS S_2_ forms. The HS S_2_ form could exhibit two short and two long Mn–Mn interactions, in which the central oxygen (O_5_) is required to be nearly at the center between Mn_A4_ and Mn_D1_. Fig. 6 shows the possible structural changes of the HS and LS S_2_ states, based on the EXAFS observation. In these models, we kept the formal oxidation state assignment of each Mn as suggested by Pantazis *et al.*^21^ and Isobe *et al.*,^47^ in which Mn^III^ is located at the Mn_D1_ in LS S_2_ form, while it is at the Mn_A4_ in HS form. Within our current knowledge, it is reasonable to think that the *S*_total_ = 1/2 being formed with anti-ferromagnetically-coupled Mn^III^ and Mn^IV^ along with two anti-ferromagnetically-coupled Mn^IV^, and *S*_total_ = 5/2 being formed with three ferro-magnetically coupled Mn^IV^ in the cubane moiety with anti-ferromagnetically-coupled Mn^III^ at Mn_A4_.

**Fig. 7:**
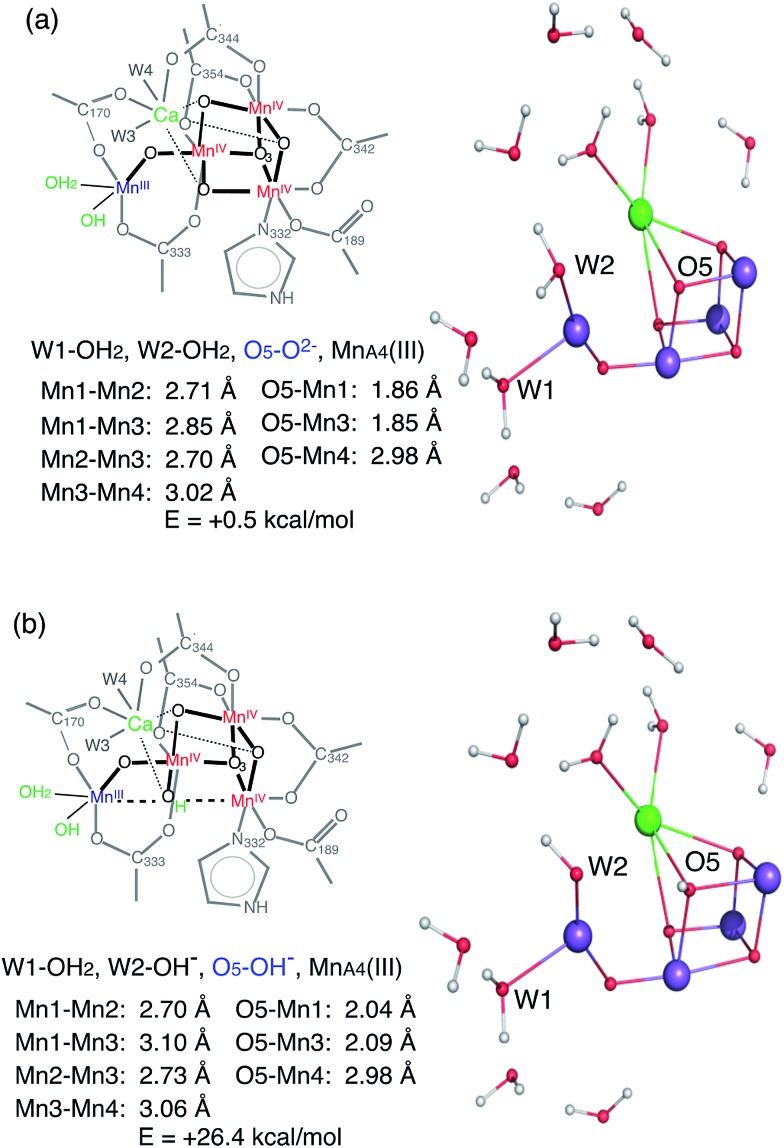
Two possible HS S_2_ models, with deprotonated (top) and protonated (bottom) O_5_, determined by QM calculations.

The XANES edge position of the HS S_2_ appears slightly lower than that of the LS form, suggesting that the effective charge density of the HS form may be lower than that of LS. This observation is also supported by the Mn Kβ_1,3_ XES results. As formal oxidation state and number of unpaired spins should be the same between HS and LS S_2_ state (although the total number of spin differs due to exchange coupling of the four Mn), one speculation is that the different protonation states of the ligand oxygen or geometry of the cluster in these two states shifts the effective charge density on Mn. If the protonation state of a ligand oxygen is different, O_5_ located between Mn_D1_ and Mn_A4_ is one possible candidate that could weaken two Mn–Mn interactions and therefore result in two long (>3.0 Å) Mn–Mn interactions. The deprotonated O_5_ in the LS S_2_ form is confirmed by EXAFS that shows all three distances, Mn_A4_–Mn_B3_, Mn_B3_–Mn_C2_, Mn_C2_–Mn_D1_, to be around ∼2.74 Å.^16^ On the contrary, if O_5_ is protonated in the HS form, both Mn_1D_–Mn_3B_ and Mn_4D_–Mn_3B_ will be elongated. The difference in oxo-bridge protonation state may affect the effective charge density on Mn. However, the S_2_ structure with protonated O_5_ in the MnIII,IV,IV,IV4 oxidation state is expected to be energetically much higher (∼25 to 30 kcal) compared to the deprotonated O_5_ (Fig. 7). This is also observed in a recent theoretical study by Krewald *et al.*^10^ Thus, this S_2_ state cannot exist as a stable form, unless there are other factors that stabilize such a structure. Hence, the difference in the geometry between the two states may be the reason for the shift in effective charge density in the HS S_2_ state.

As shown in Fig. 3a, the EXAFS spectra of HS and LS S_2_ states and the S_3_ state are all different. This implies that the atomic distances of these three forms are different. The structural model of HS S_2_ state proposed from the QM calculations is very similar to one of the two structural models proposed by us for the S_3_ state in a previous study.^16^ Studies by Cox *et al.* with EPR and QM calculations^20^ and Siegbahn *et al.* with earlier QM calculations,^52^ suggest that the S_2_ LS and S_3_ structure share the same Mn_3_Ca geometry with an open-cubane structure, except for an additional water molecule ligated in the open coordination site of Mn_D1_ in the S_3_ state. Our group has proposed a S_3_ state structure to comprise a closed-cubane structural motif, based on the EXAFS studies (Fig. 6) that showed an elongation of Mn–Mn distances within the cubane-motif. The rationale of our proposal is from inorganic model compound studies where the elongation of metal–metal distances is observed when the cubane is formed.^74^,^75^ A similar elongation of the metal–metal distances is visible in the S_3_ EXAFS spectrum (*i.e.* while all three di-μ-oxo Mn–Mn distances are ∼2.74 Å in the S_2_ state, it is more distributed over the range of 2.72–2.82 Å in the S_3_ state).^16^

In the current study, we observed that the HS S_2_ EXAFS is different from that of S_3_ state, suggesting that the geometries of HS S_2_ and S_3_ state structures are likely different. One possibility is that, as depicted in Fig. 6, in LS S_2_ O_5_ is bound to Mn_A4_, while in HS S_2_ it is more or less equidistant from Mn_A4_ and Mn_D1_. In S_3_ state, the O_5_ position is shifted to Mn_D1_. However, an uncertainty remains if the S_3_ state has a large heterogeneity (EPR active and EPR silent species), as suggested by Boussac *et al.*^45^ and Cox *et al.*^20^ based on EPR studies. Then the EXAFS spectrum under our experimental condition could be a mixture of the EPR active and the EPR silent species. Further studies of this potential heterogeneity are necessary. Also, we cannot eliminate the possibility of the inserted water model suggested by Cox *et al.*^20^ and Siegbahn *et al.*,^52^ if the elongation of the metal–metal distances in the S_3_ state occurs by the expansion of the open-cubane moiety due to the effect of newly inserted water into the open Mn1 site (Fig. 6).

### Transition process between the S_1_, S_2_, to S_3_ states

**Fig. 8:**
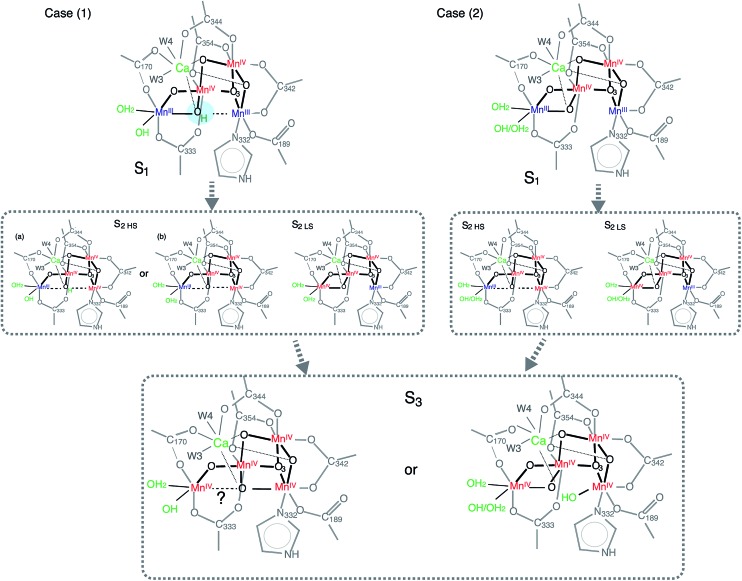
Structural model showing two possible reaction pathways during S_1_–S_2_–S_3_ transitions with protonated O_5_ (case 1) and deprotonated O_5_ (case 2).

Currently, the radiation-damage-free dark state structure published by Suga *et al.* with 1.95 Å resolution^4^ serves as the most reliable foundation for considering possible distance changes in the higher S-states. Mn–Mn distances and number of interactions in the crystal structure matches reasonably well with the structural parameters obtained from earlier EXAFS studies of the S_1_ state (*i.e.* there are three short 2.7–2.8 Å Mn–Mn interactions and one long ∼3.3 Å Mn–Mn interaction in addition to three Mn–Ca interactions).^16^,^59^,^60^ One of the remaining uncertainties in the S_1_ structure, however, is the protonation state of O_5_. Suga *et al.* suggest O_5_ to be protonated, based on the long Mn_A4_–O_5_ and Mn_D1_–O_5_ distances^4^ in which the O_5_ position was obtained from the omit map. However, there is another explanation that proposes a deprotonated O_5_ in the S_1_ state. In the following section, we discuss these two possibilities, case (1) for protonated O_5_ and case (2) for deprotonated O_5_ in the S_1_ state, in relation to the S_2_ state formation (Fig. 8).

In case (1), the O_5_ proton needs to move away from the OEC upon S_1_ to S_2_ (*via* 200 K illumination or RT laser flash) transition, as it is most likely that O_5_ is deprotonated in the LS S_2_ form. If the protonation state is different between HS and LS S_2_ states, one possible reason for this to occur is that O_5_ is protonated in the S_1_ state, and remains as it is in the HS S_2_ (case (1), S_2_ HS (a) in Fig. 8). Small structural or chemical changes that are required for the proton motion could be prohibited under the illumination condition at 140 K with NIR illumination while going to the ‘native’ S_2_ state may require an illumination at higher temperature. As previously discussed, the S_2_ structure with protonated O_5_ is energetically much higher than the deprotonated one (Fig. 7), which makes this model highly unlikely. Moreover, it is difficult to rationalize an observed reversibility between LS S_2_ and HS S_2_ form with the O_5_-protonated model. Therefore, the O_5_ proton in the S_1_ state needs to move to a nearby ligand in the S_1_ to HS S_2_ transition (case (1), S_2_ HS (b) in Fig. 8). The EXAFS curve fitting results in this study suggest that there could be two elongated Mn–Mn interactions longer than 3 Å, and two di-μ-oxo bridged Mn–Mn interactions at ∼2.7 Å. Such a structure is different from the one proposed from the theoretical studies.^21^,^47^ Although we cannot completely rule out a model that contains three 2.7 Å Mn–Mn and one 3.3 Å Mn–Mn, a complete cubane formation at HS S_2_ state seems to be less likely from the current EXAFS data due to decreased heterogeneity around 2.7 Å interactions. The structural differences in HS and LS S_2_ states could be reasoned by O_5_ position moving closer to Mn_1D_ upon oxidation of Mn_1D_ from Mn^III^ to Mn^IV^ during the S_1_ to HS S_2_ state transition, but Mn_1D_–O_5_ is weakly bound with a distance longer than 2 Å. In case (2) in which O_5_ is deprotonated in the S_1_ state, a similar argument to case (1) is applicable (Fig. 8).

The question arises whether the HS S_2_ state populated by 140 K NIR illumination, that we observed in this study, is the same as the S_2_ state with enhanced *g* = 4 signals that is observed at higher temperature illumination (200 K illumination or laser flash at room temperature) under certain buffer conditions (*e.g.* with sucrose buffer) or additional chemical treatments (*e.g.* with a substitution of Ca^2+^ by Sr^2+^). Moreover, whether the HS and LS S_2_ species is populated under physiological conditions and such heterogeneity plays a role in the S-state advancement and the catalytic reaction still remains unanswered. While this question requires the room temperature study, here we speculate if the *g* = 4 species made under different conditions are always the same. It has been suggested that any changes that disturb the hydrogen bond network around the OEC influence the electronic properties of Mn_A4_ through the W1 and/or W2 ligands that are ligated to this Mn.^44^ There is a trend that any changes in the electron donating effect in ligands of Mn_A4_ favor this site to have a lower Mn oxidation state (*i.e.* Mn^III^).^44^ The high-resolution crystal structure of the *T. vulcanus* PSII^3^ has shown that the hydrogen bond network around OEC is extended from W1 and W2 of Mn_A4_ to D1–D61 (Fig. S7†). Pokhrel *et al.* suggested that this hydrogen bonding network of W1 and W2 determines the equilibrium between the *g* = 2 and *g* = 4 forms of the OEC in the S_2_ state.^44^ As a consequence, we can speculate that the HS S_2_ EPR signal around the *g* = 4 region changes and its exact *g*-value (*i.e.* the zero-field splitting) is highly affected by such effects. The fact that we observe changes in HS S_2_ state from LS S_2_ state implies that the OEC structure that causes the *g* ∼ 4 EPR signal, whether by buffer contents or chemical treatments, is different from that of the *g* = 2 species. A question still remains if all the *g* = 4 species observed in the EPR spectra represents the same structure. This may not be the case if some of the *g* = 4 signals under certain conditions are not from the *S*_total_ = 5/2 ground state, but from the excited state of a different ground state spin configuration, thus these signals may represent other ground state spin configurations. For example, the temperature-dependent EPR signal from a weakly-coupled Mn(iii/iv) dimer core has been reported in a model system.^76^ In this case, the 16-line spectrum of the ground state (*S*_total_ = 1/2) and the *g* ∼ 5 spectrum from the excited state (*S*_total_ = 3/2) are observed. No observation of temperature dependence when going from S_2_-*g*2 to S_2_-*g*4 EPR signals in PSII suggest that both the signals studied here arise from the ground state. Also, since the proposed models available are based on EPR measurements that are all measured at low temperature, the S_2_ species that exist in room temperature still remains unknown.

In the current study, we use spinach PSII to investigate the S_2_-*g*2 and S_2_-*g*4 states. Unlike spinach PSII in which the S_2_-*g*4 signal is observed under certain buffer conditions, there are additional EPR signals along with the S_2_-*g*4 signal in *Synechococcus* PSII wild type. In these samples, *g* = 6 to 10 signals are also observed when samples are IR illuminated, and the pure *g* = 4 signal is only observed when the native Ca^2+^ or Cl^–^ is substituted. Nevertheless, the intensity of these low EPR field signals are weak, and it suggests that S_2_-*g*2 state is the dominant species in the wild type. The reason for such species dependence is not known, as the crystal structure is only available for the *Synechococcus* PSII. However, it is likely due to small differences in the hydrogen-bonding network that extends from W1 and W2 of the OEC to the water channel leads to subtle differences in the electronic structure.

## Conclusions

We have investigated the structure and the electronic structure of the two spin-isomers in the PSII S_2_ intermediate states. The XAS data suggests different structural configurations for the HS and LS S_2_ states. It also suggests that their structures are different from the subsequent S_3_ state. Whether the HS S_2_ state serves as an intermediate state between the LS S_2_ and the S_3_ state as proposed from theoretical modeling is still an open question, and the high-resolution crystal structure of these intermediate states, possibly at the room temperature is necessary to resolve it. Despite such noticeable structural differences during the catalytic pathway due to the likely modification of the hydrogen-bonding network, the O_2_ evolution activity remains similar for both spin forms. This implies a certain flexibility of the OEC in its geometric and electronic structure under physiological conditions, although one state may be more preferable than the other.

## Supplementary Material

Supplementary information
