# Supplementary material for: Structural changes correlated with magnetic spin state isomorphism in the S2 state of the Mn4CaO5 cluster in the oxygen-evolving complex of photosystem II
Source: Chem Sci. 2016 May 9;7(8):5236–48. doi: 10.1039/c6sc00512h (PMC5201215; doi:10.1039/c6sc00512h)
Supplement: Supplementary file 1 [file SC-007-C6SC00512H-s001.pdf]

Electronic Supplementary Material (ESI) for Chemical Science.
This journal is © The Royal Society of Chemistry 2016

Electronic Supporting Information

Structural Changes Correlated with Magnetic Spin State Isomorphism in the S_2 State of the Mn_4CaO_5 Cluster in the Oxygen-Evolving Complex of Photosystem II

Ruchira Chatterjee,[a,#] Guangye Han,[a,#,§] Jan Kern[a,b], Sheraz Gul[a], Franklin D. Fuller [a], Anna Garachtchenko[a], Iris Young[a], Tsu-Chien Weng[c,¶], Dennis Nordlund[c], Roberto Alonso-Mori[b], Uwe Bergmann[b,c], Dimosthenis Sokaras[c], Makoto Hatakeyama[d], Vittal K. Yachandra[a,*], Junko Yano[a,*]

[a] Molecular Biophysics and Integrated Bioimaging Division, Lawrence Berkeley National Laboratory, Berkeley, CA, USA

[b] LCLS, SLAC National Accelerator Laboratory, Menlo Park, CA

[c] Center for High Pressure Science &Technology Advanced Research, Shanghai, China.

[d] SSRL, SLAC National Accelerator Laboratory, Menlo Park, CA

[e] PULSE, SLAC National Accelerator Laboratory, Menlo Park, CA

[f] RIKEN Research Cluster for Innovation, Wako, Saitama, Japan

EXAFS curve-fitting procedure

Curve fitting was performed with Artemis and IFEFFIT software using *ab initio*-calculated phases and amplitudes from the program FEFF 8.2.[1,2] These *ab initio* phases and

$$\chi(k) = S_0^2 \sum_j \frac{N_j}{kR_j^2} f_{eff_j}(\pi,k,R_j) e^{-2\sigma_j^2 k^2} e^{-2R_j/\lambda_j(k)} \sin(2kR_j + \phi_{ij}(k)) \qquad \text{(S1)}$$

amplitudes were used in the EXAFS equation (S1):

The neighboring atoms to the central atom(s) are divided into *j* shells, with all atoms with the same atomic number and distance from the central atom grouped into a single shell. Within each shell, the coordination number N_j denotes the number of neighboring $f_{\text{eff j}}$ (π,k,R_j) atoms in shell *j* at a distance of R_j from the central atom. is the *ab initio* amplitude function for shell *j*, and the Debye-Waller term $e^{-2\sigma j2k2}$ accounts for damping due to static and thermal disorder in absorber-backscatterer distances. The mean free path term $e^{-2R\text{j}/\lambda\text{j}(k)}$ reflects losses due to inelastic scattering, where $\lambda_j(k)$ is the electron mean free path. The oscillations in the EXAFS spectrum are reflected in the sinusoidal term, $\sin(2kR_j + \varphi_{ij}(k))$ where $\varphi_{ij}(k)$ is the *ab initio* phase function for shell *j*. S_0^2 is an amplitude reduction factor due to shake-up/shake-off processes at the central atom(s). The EXAFS equation was used to fit the experimental data using *N*, *R*, and the EXAFS Debye-Waller factor (σ^2) as variable parameters. E_0 was defined as 6545.0 eV and the S_0^2 value was fixed to 0.85 for the energy (eV) to wave vector (k, Å^{-1}) axis conversion. Note that fits are comparisons to proposed models thus the N parameter is fixed, while the Debye-Waller is varied to get the best fit. Fixing the Debye-Waller factor could bias one model more than the other. R%, the R-factor goodness of fit is:

$$R\% = \sum_1^N |(1/s_i)^2[\chi^{expt}(k_i) - \chi^{calc}(k_i)] \qquad \text{(S2)}$$

1*i* where *N* is the total number of data points collected, $\chi^{expt}(k_i)$ is the experimental EXAFS amplitude at point *i*, and $\chi^{calc}(k_i)$ is the theoretical EXAFS amplitude at point *i*. The normalization factor s_i is given by:

$$1/s_i = k_i^3 / \sum_j^N k_j^3 |\chi_j^{\text{exptl}}(k_j)| \qquad \text{(S3)}$$

Table S1. Mn XANES pre-edge peak fit areas of (a) LS S_2 and (b) HS S_2 states.

	Area under curve 1 (6540 eV)	Area under curve 2 (6541 eV)	Area under curve 3 (6542 eV)	Total area
LS S_2 state	0.043	0.130	0.072	0.245
HS S_2 state	0.024	0.141	0.064	0.229

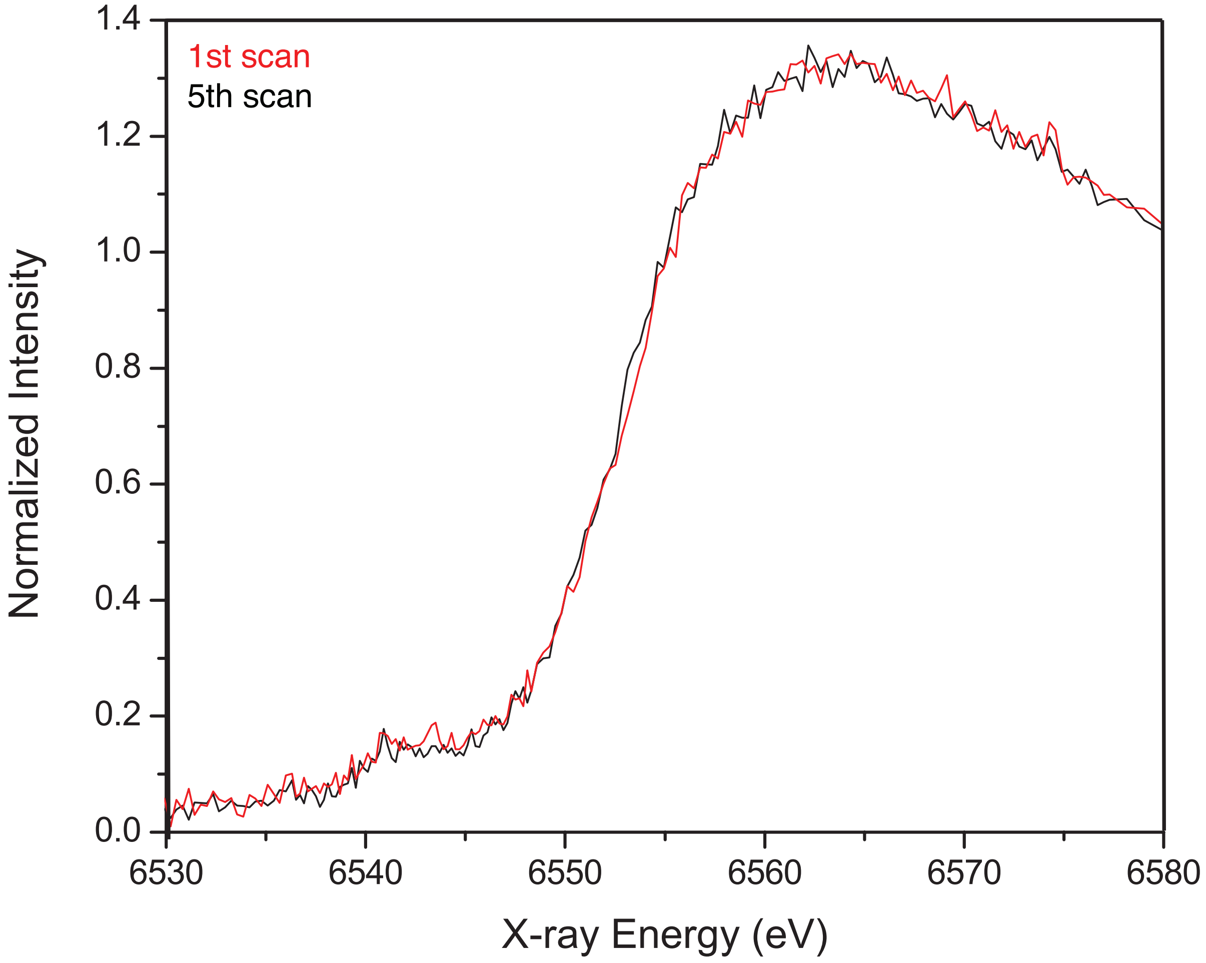

Figure S1. Mn XAS spectra of S_2 140 K NIR illuminated 1st scan (red) and 5th scan (black). Respective scans from six sample spots were added for each spectrum shown here.

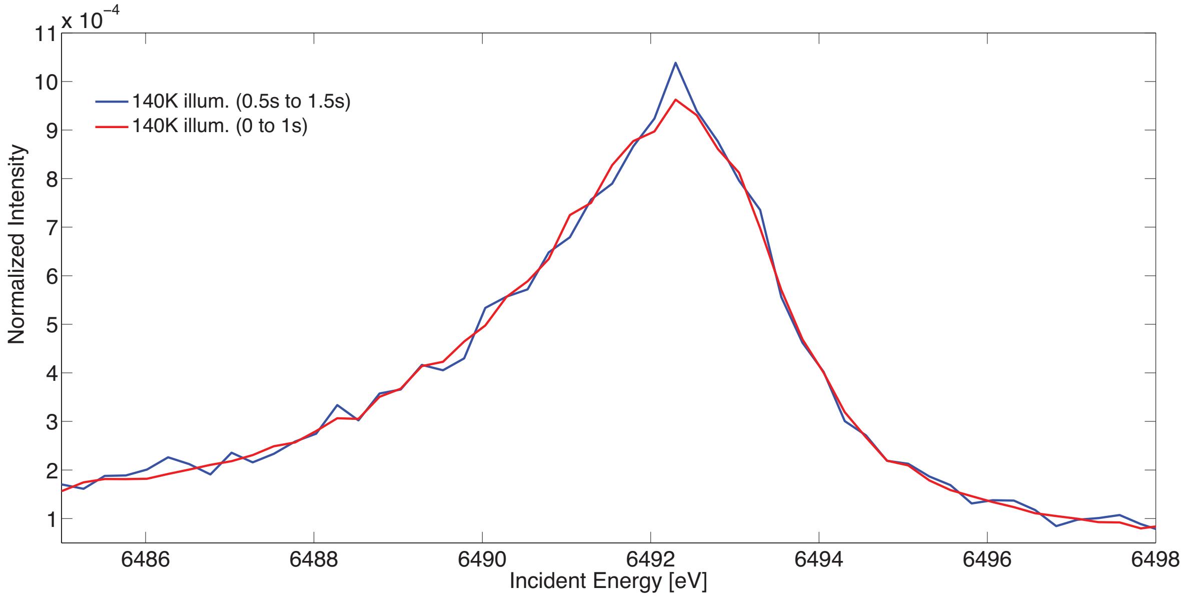

Figure S1. Mn XES spectra of S_2 140 K NIR illuminated bin 11-30 (0.5s -1.5s) (blue) and first 20 bins (0- 1s) (red).

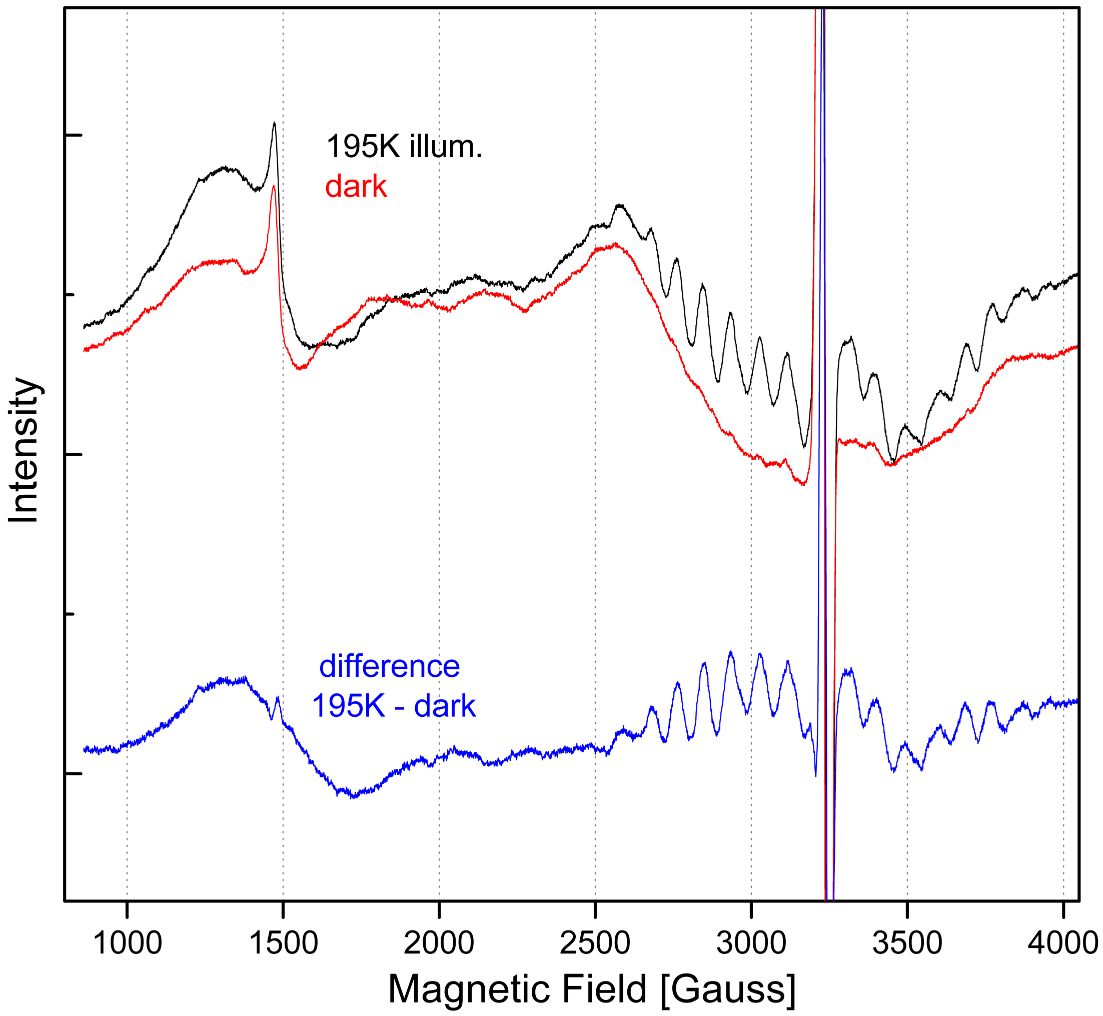

Figure S3. EPR spectra of PSII samples in sucrose illuminated for 10 mins at 195 K (black) and dark (red) EPR spectra. The difference spectrum (blue) is between the spectra after illumination and the spectra of the same dark-adapted sample. The spectra are the difference between the spectra after illumination and the spectra of the same dark-adapted sample. Spectrometer condition: microwave frequency, 9.22 GHz; field modulation amplitude, 32 G at 100 KHz; microwave power, 20 mW. The spectra are collected at 8K.

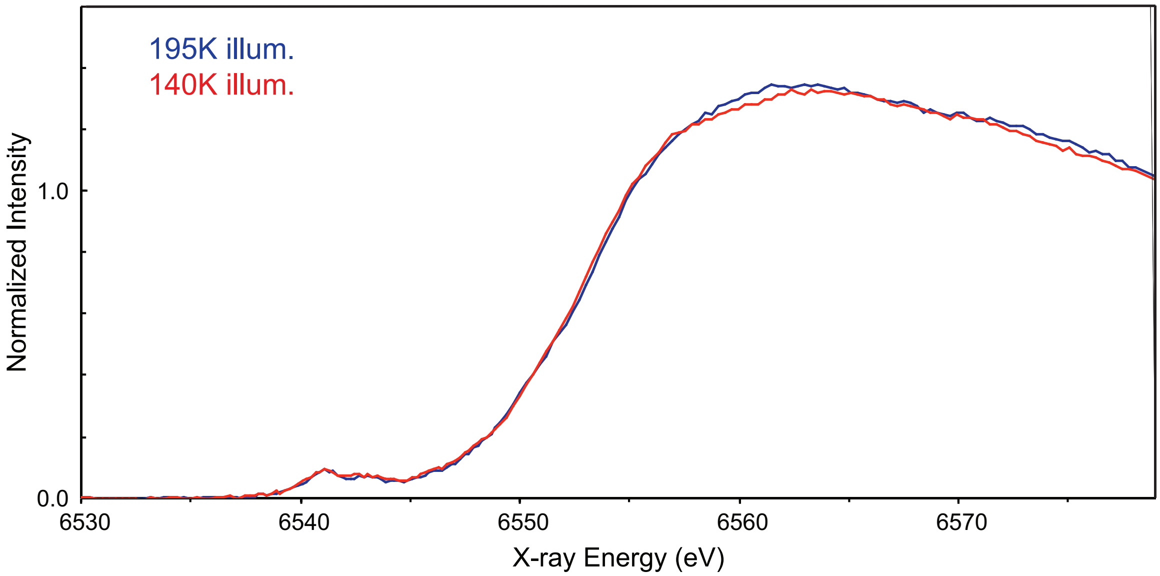

Figure S4. Mn XAS spectra of S_2 195 K illuminated (blue) and 140 K NIR illuminated S_2 states (red).

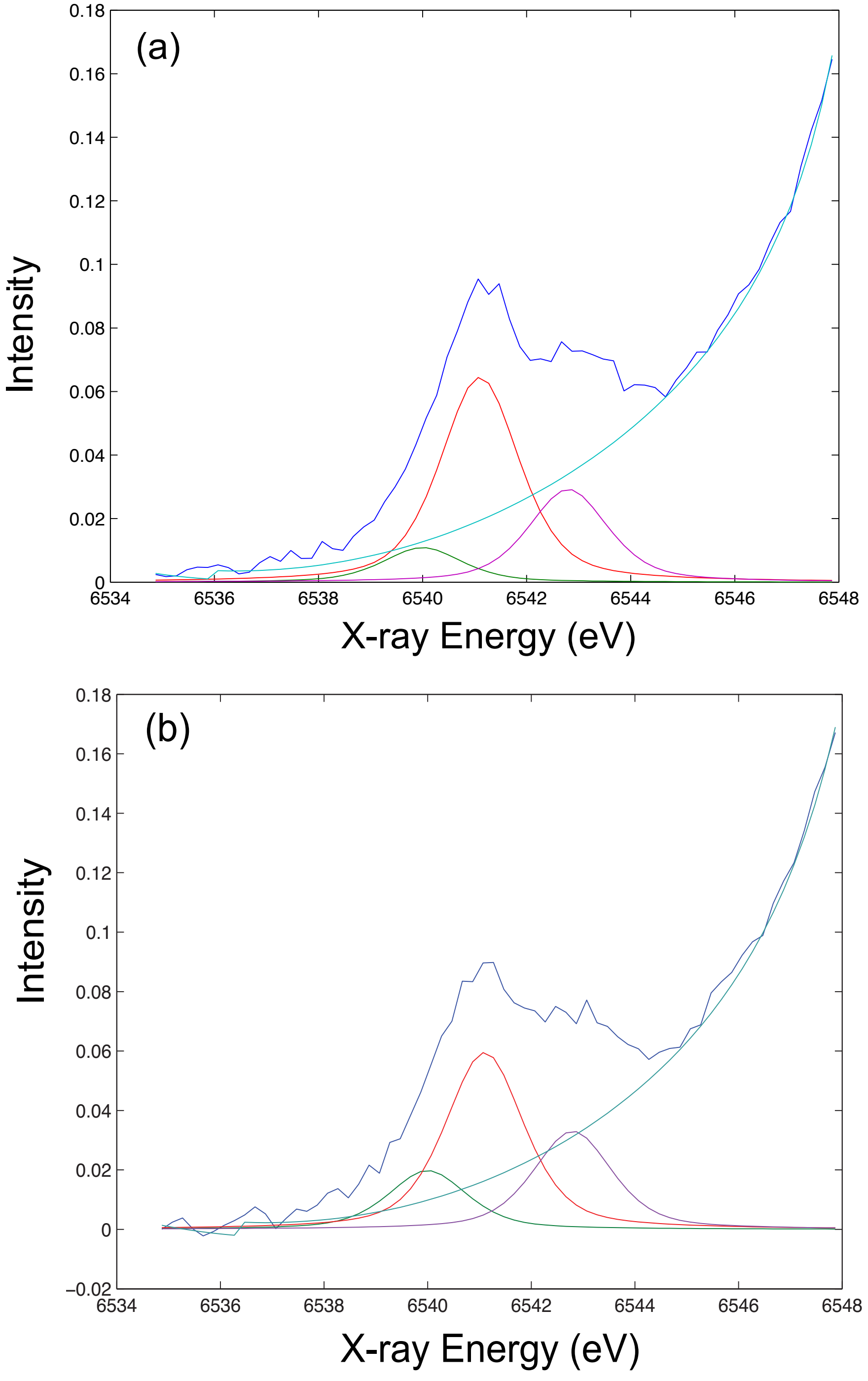

Figure S5. Mn XANES pre-edge peak fit of (a) LS S_2 and (b) HS S_2 states.

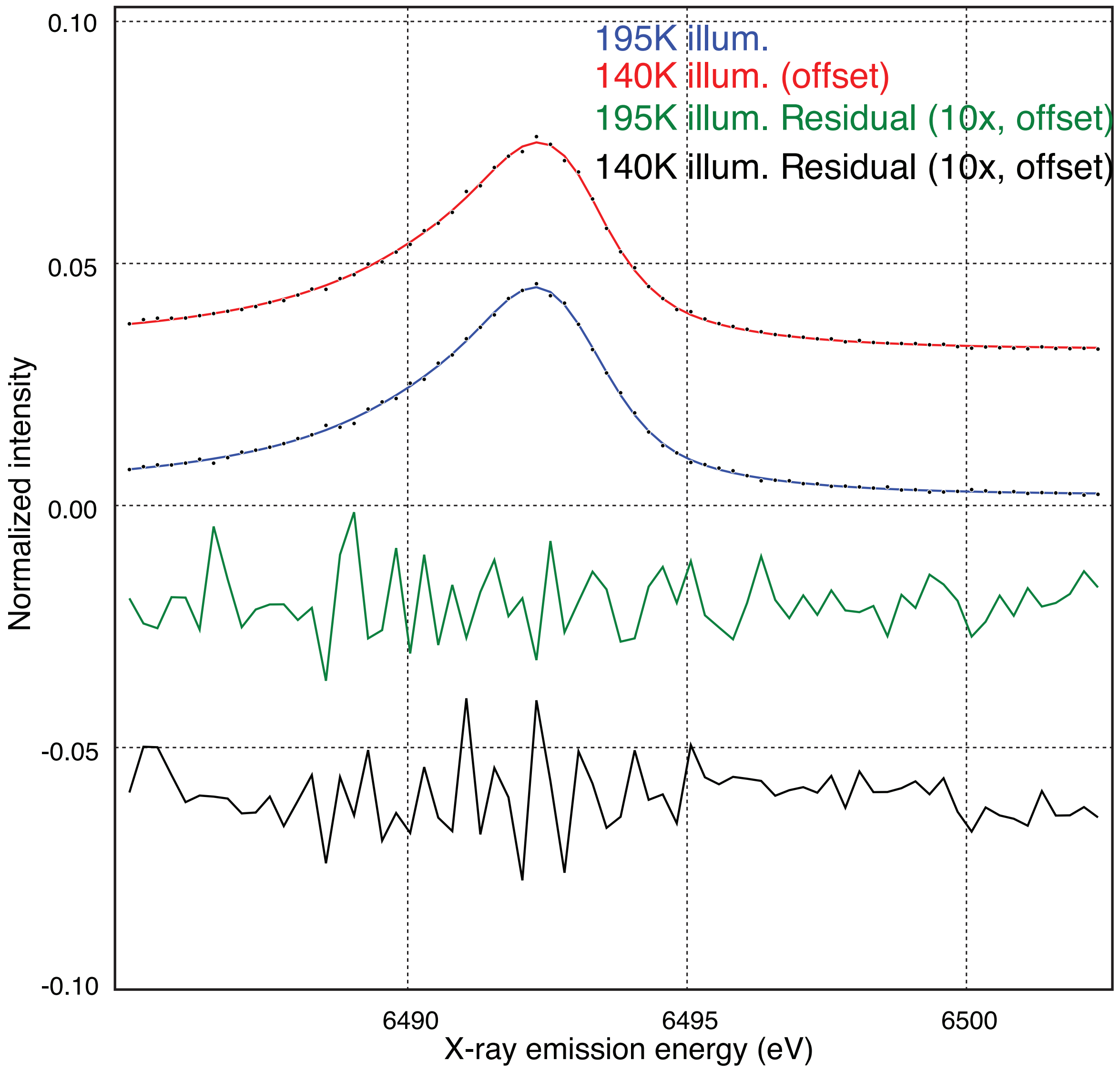

Figure S6. K$\beta_{1,3}$ XES spectra of 195K (blue) and 140 NIR (red) illuminated S_2 states. Raw data (dots) and the smoothed (lines) of both LS and HS states. The residual plots of the LS (green) and HS (black) S_2 states show that the smoothed spectra are a good fit of the raw data.

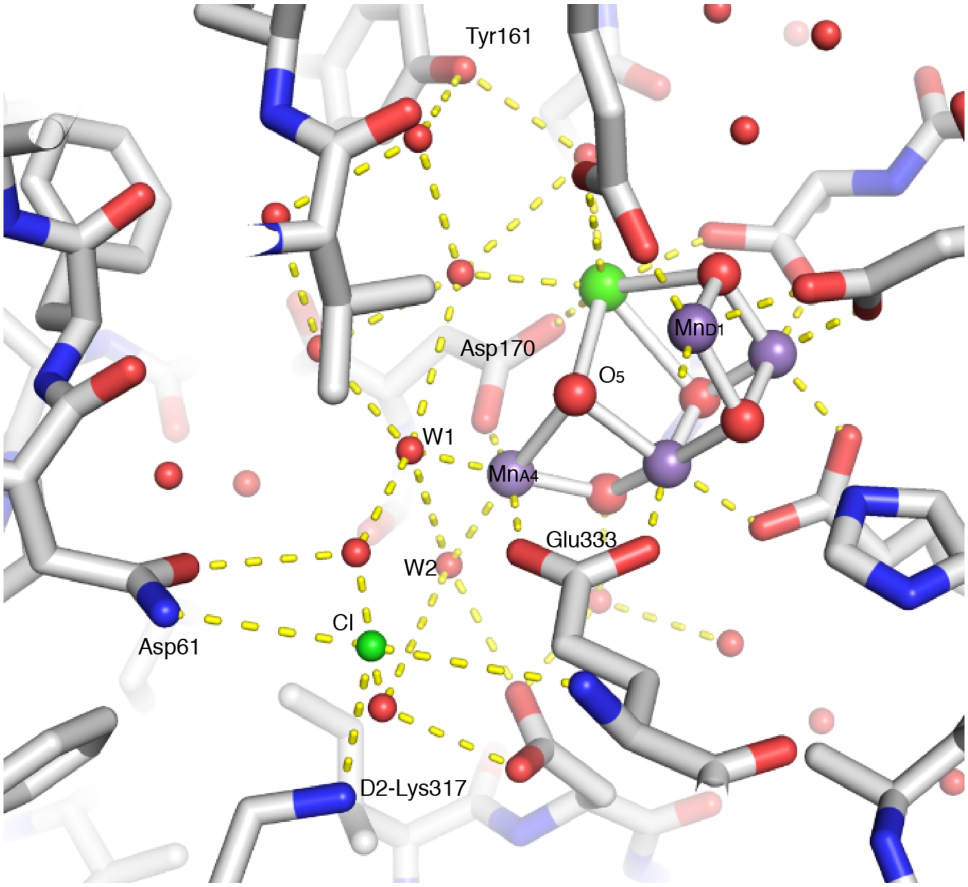

Figure S7. The hydrogen bonding network that extends from W1 and W2 that are ligated to the Mn_4CaO_5 cluster.[3]

DFT coordinates of the high-layer structure shown in Figure 7a

Mn	190.419998	97.260002	83.971001
Mn	191.869003	99.417000	83.207001
Mn	189.266006	99.855003	83.773003
Mn	187.438995	100.379997	86.115997
Ca	191.112000	99.200996	86.570000
O	191.873993	98.151001	84.499001
O	190.873993	100.432999	84.285004
O	190.240005	98.592003	82.686996
O	188.488998	101.053001	84.869003
O	189.337006	98.416000	84.936996
C	189.419006	102.188004	89.382004
H	190.067993	103.065002	89.444000
H	189.839996	101.453003	90.081001
C	189.552994	101.524002	88.024002
O	190.653000	101.494003	87.439003
O	188.533997	100.860001	87.603996
C	192.572998	93.980003	87.802002
H	191.962997	94.012001	88.709999
H	193.438995	94.636002	87.928001
C	191.830994	94.346001	86.522003
H	192.563995	94.292000	85.711998
H	191.033997	93.629997	86.294998
C	191.237000	95.705002	86.288002
O	191.235992	96.644997	87.097000
O	190.781998	95.803001	85.078003
C	185.692993	94.306000	83.174004
H	185.281998	94.788002	84.056999
H	185.854996	93.272003	83.481003
C	187.007996	94.975998	82.956001
N	187.945007	94.565002	82.033997
H	187.813995	93.831001	81.341003
C	189.080994	95.253998	82.208000
H	189.988007	95.086998	81.652000
N	188.925995	96.125000	83.190002
C	187.636993	95.970001	83.658997
H	187.270004	96.553001	84.490997
C	185.100006	98.751999	81.431999
H	186.056000	98.393997	81.050003
H	184.987000	99.778000	81.068001
C	185.171005	98.734001	82.955002
H	184.309006	99.216003	83.419998
H	185.192001	97.704002	83.332001
C	186.421005	99.280998	83.596001
O	187.503998	99.347000	82.963997

O	186.296005	99.539001	84.844002
C	189.718002	98.241997	76.598999
H	189.177994	97.509003	75.999001
H	190.783997	98.047997	76.439003
C	189.408997	98.057999	78.046997
N	188.218002	97.523003	78.511002
C	188.207993	97.504997	79.845001
H	187.408005	97.115997	80.456001
N	189.352005	98.019997	80.260002
H	189.610992	98.179001	81.255997
C	190.117996	98.370003	79.171997
H	191.100998	98.799004	79.276001
C	193.432999	96.075996	81.094002
H	192.787994	95.703003	80.290001
H	193.778000	95.205002	81.653999
C	192.589996	96.931999	82.004997
O	192.854004	98.172997	82.073997
O	191.684006	96.338997	82.670998
C	195.371994	100.612000	84.882004
H	195.804001	100.050003	85.711998
C	195.597000	102.112000	85.061996
H	195.033997	102.692001	84.321999
H	195.255005	102.414001	86.057999
H	196.656006	102.365997	84.983002
C	193.903000	100.313004	84.861000
O	193.227005	100.211998	85.876999
O	193.460007	100.143997	83.639000
C	191.628998	103.098000	80.143997
H	192.509995	102.628998	79.703003
H	191.914001	103.362999	81.169998
C	190.445999	102.122002	80.238998
H	189.494995	102.649002	80.322998
H	190.384995	101.481003	79.349998
C	190.556000	101.203003	81.444000
O	191.677994	100.649002	81.695000
O	189.507004	101.039001	82.142998
C	192.552002	108.153000	84.084999
H	192.479996	108.474998	85.129997
H	191.554001	107.810997	83.793999
C	193.585007	107.022003	83.970001
H	193.707993	106.685997	82.935997
H	194.569000	107.373001	84.297997
C	193.270996	105.811996	84.852997
H	194.095001	105.095001	84.776001
H	193.188004	106.132004	85.902000
N	192.039993	105.128998	84.440002

H	191.311996	105.717003	84.042999
C	191.593002	103.996002	85.008003
N	192.354004	103.362999	85.912003
H	193.126007	103.859001	86.351997
H	191.947998	102.595001	86.439003
N	190.421005	103.475998	84.644997
H	189.888000	103.822998	83.845001
H	190.097000	102.587997	85.017998
H	187.582001	99.809998	90.626999
O	187.572998	99.114998	89.944000
H	187.621994	98.242996	90.426003
H	184.026993	101.000000	88.875999
O	184.078995	100.544998	88.010002
H	183.201996	100.661003	87.601997
H	186.554993	104.876999	84.739998
O	186.962997	103.997002	84.674004
H	186.139008	103.470001	84.477997
H	192.845001	98.106003	88.769997
O	193.042007	98.439003	87.876999
H	193.779007	99.075996	87.991997
H	190.048004	97.820000	89.014999
O	189.957993	98.711998	88.626999
H	189.108002	99.041000	88.988998
H	191.186996	96.488998	90.247002
O	190.442001	96.144997	89.722000
H	190.800003	96.053001	88.811996
H	193.229004	97.206001	85.084999
O	193.919998	96.792999	85.636002
H	193.582993	96.987999	86.531998
H	186.815994	99.255997	88.442001
O	186.481995	99.204002	87.507004
H	185.511993	99.301003	87.539001
H	185.462006	102.333000	85.776001
O	185.938004	101.950996	86.550003
H	185.207993	101.593002	87.106003
H	188.809998	96.373001	90.598999
O	188.104996	96.816002	91.101997
H	187.529999	96.126999	91.470001
H	188.477997	102.588997	82.349998
O	188.391006	103.556999	82.310997
H	187.787003	103.755997	83.056000
H	187.434998	97.231003	77.932999

DFT coordinates of the high-layer structure shown in Figure 7b

Mn	190.492004	97.207001	83.913002
Mn	191.834000	99.448997	83.236000
Mn	189.209000	100.018997	83.705002
Mn	187.440994	100.448997	86.164001
Ca	191.117004	99.254997	86.621002
O	191.850006	98.154999	84.515999
O	190.854996	100.462997	84.289001
O	190.246994	98.585999	82.706001
O	188.455002	101.194000	84.676003
O	189.315002	98.421997	85.047997
C	189.507996	102.255997	89.428001
H	190.136993	103.143997	89.530998
H	189.931000	101.514999	90.119003
C	189.699997	101.633003	88.056999
O	190.863998	101.511002	87.594002
O	188.695999	101.126999	87.464996
C	192.576004	93.980003	87.803001
H	191.960999	94.013000	88.708000
H	193.442993	94.635002	87.932999
C	191.841995	94.341003	86.518997
H	192.578003	94.277000	85.710999
H	191.042999	93.625999	86.293999
C	191.251999	95.697998	86.266998
O	191.225998	96.648003	87.055000
O	190.813004	95.773003	85.039001
C	185.690002	94.303001	83.172997
H	185.283997	94.795998	84.052002
H	185.845001	93.269997	83.487999
C	187.009003	94.960999	82.946999
N	187.938995	94.555000	82.017998
H	187.800995	93.825996	81.320999
C	189.078003	95.237999	82.190002
H	189.977005	95.066002	81.624001
N	188.936005	96.105003	83.178001
C	187.645004	95.947998	83.650002
H	187.246002	96.512001	84.480003
C	185.100998	98.755997	81.421997
H	186.054001	98.408997	81.022003
H	184.975006	99.782997	81.066002
C	185.199005	98.728996	82.941002
H	184.354004	99.221001	83.427002
H	185.205994	97.699997	83.319000
C	186.462006	99.263000	83.570000
O	187.542999	99.345001	82.903999

O	186.386002	99.476997	84.811996
C	189.716003	98.241997	76.600998
H	189.175003	97.508003	76.000999
H	190.781006	98.045998	76.442001
C	189.404999	98.059998	78.049004
N	188.212006	97.532997	78.515999
C	188.201004	97.518997	79.848999
H	187.397995	97.139999	80.460999
N	189.348999	98.028000	80.264000
H	189.608994	98.189003	81.260002
C	190.115997	98.370003	79.172997
H	191.102997	98.792999	79.271004
C	193.429993	96.082001	81.101997
H	192.776001	95.714996	80.303001
H	193.772003	95.209999	81.663002
C	192.610992	96.959999	82.012001
O	192.871994	98.182999	82.098000
O	191.690002	96.356003	82.686996
C	195.367004	100.617996	84.872002
H	195.804001	100.069000	85.709000
C	195.578995	102.121002	85.037003
H	195.022003	102.689003	84.283997
H	195.223007	102.431999	86.025002
H	196.638000	102.378998	84.968002
C	193.903000	100.304001	84.852997
O	193.237000	100.160004	85.866997
O	193.445999	100.157997	83.630997
C	191.623993	103.101997	80.142998
H	192.492996	102.621002	79.692001
H	191.923996	103.365997	81.165001
C	190.427002	102.146004	80.250999
H	189.485001	102.686996	80.360001
H	190.337997	101.508003	79.362000
C	190.552994	101.227997	81.449997
O	191.660995	100.669998	81.709000
O	189.488998	101.078003	82.148003
C	192.552994	108.155998	84.086998
H	192.477997	108.480003	85.132004
H	191.556000	107.810997	83.795998
C	193.587006	107.026001	83.977997
H	193.709000	106.683998	82.945999
H	194.572006	107.382004	84.301003
C	193.278000	105.822998	84.871002
H	194.102005	105.105003	84.795998
H	193.197998	106.150002	85.917000
N	192.044998	105.138000	84.466003

H	191.322998	105.725998	84.056000
C	191.580994	104.028000	85.066002
N	192.332993	103.399002	85.978996
H	193.123993	103.892998	86.387001
H	191.903000	102.684998	86.566002
N	190.397003	103.526001	84.713997
H	189.856995	103.901001	83.934998
H	190.042999	102.648003	85.083000
H	187.701996	99.893997	90.676003
O	187.684998	99.223000	89.968002
H	187.662003	98.346001	90.428001
H	184.033005	101.049004	88.908997
O	184.039993	100.586998	88.043999
H	183.160995	100.761002	87.666000
H	186.494003	104.846001	84.802002
O	186.959000	103.997002	84.713997
H	186.164993	103.424004	84.528999
H	192.834000	98.098999	88.779999
O	193.054993	98.408997	87.883003
H	193.783005	99.054001	88.007004
H	190.031006	97.825996	89.004997
O	189.970001	98.727997	88.637001
H	189.136993	99.079002	89.031998
H	191.179993	96.504997	90.248001
O	190.451996	96.137001	89.714996
H	190.820007	96.054001	88.811996
H	193.225998	97.240997	85.125999
O	193.919998	96.789001	85.639000
H	193.617996	96.973999	86.551003
H	187.059006	99.359001	88.239998
O	186.953995	99.177002	87.282997
H	188.473999	98.045998	85.362000
H	185.371002	102.239998	85.820000
O	185.867004	101.849998	86.575996
H	185.162003	101.403000	87.125999
H	188.785004	96.389000	90.589996
O	188.104996	96.848000	91.109001
H	187.509003	96.170998	91.463997
H	188.337006	102.615997	82.394997
O	188.294998	103.581001	82.301003
H	187.729004	103.834000	83.059998
H	187.423996	97.245003	77.940002

References:

(1) Newville, M. *Journal Of Synchrotron Radiation* **2001**, *8*, 322.
(2) Rehr, J. J.; Albers, R. C. *Reviews of Modern Physics* **2000**, *72*, 621.
(3) Suga, M.; Akita, F.; Hirata, K.; Ueno, G.; Murakami, H.; Nakajima, Y.; Shimizu, T.; Yamashita, K.; Yamamoto, M.; Ago, H.; Shen, J.-R. *Nature* **2015**, *517*, 99.
